# Designed synthesis of a “One for Two” hydrophilic magnetic amino-functionalized metal-organic framework for highly efficient enrichment of glycopeptides and phosphopeptides

**DOI:** 10.1038/s41598-017-01341-y

**Published:** 2017-04-25

**Authors:** Yiqin Xie, Chunhui Deng

**Affiliations:** Department of Chemistry and Institutes of Biomedical Sciences, Collaborative Innovation Center of Genetics and Development, Fudan University, Shanghai, 200433 China

## Abstract

Highly efficient enrichment of glycopeptides or phosphopeptides from complex biological samples is indispensable for high-throughput mass spectrometry analysis. In this study, for the first time, a “one for two” hydrophilic magnetic amino-functionalized metal-organic framework (MOF) was designed and synthesized for selective enrichment of both glycopeptides and phosphopeptides. A well-known solvo-thermal reaction was adopted to prepare a magnetic core Fe_3_O_4_, followed by self- polymerization of dopamine, creating a polydopamine (PDA) onto Fe_3_O_4_. Thanks to the hydroxyl and amino group of PDA, Zr^3+^ was easily adhered to the surface, inducing the following one-pot MOF reaction with amino ligand. After characterization of the as-prepared MOFs (denoted as Fe_3_O_4_@PDA@UiO-66-NH_2_), its ultrahigh surface area, excellent hydrophilicity and strong magnetic responsiveness were highly confirmed. Based on hydrophilic interaction, it was applied to glycopeptide enrichment, while based on strong binding between Zr and phosphopeptides, it was applied to phosphopeptide enrichment, both exhibiting excellent performance in standard proteins and human serum with high sensitivity and selectivity. These results showed the as-prepared MOFs had great potential in proteomics research.

## Introduction

Protein glycosylation and phosphorylation, two of the most important post-translational modifications (PTMs), play a critical role in many biological processes, such as signal transduction^1^, cell-cell interaction^2^, cell adhesion^3^, and so on. As abnormal phosphorylation or glycosylation serves as the biomarkers for several types of cancers and diseases^4, 5^, it is of great importance to identify the certain sites, and the corresponding proteins. To dates, Mass spectrometry (MS) has become one of the most common tools to identify glycosylation and phosphorylation because of its high throughput, fast speed and high sensitivity^6–8^. Unfortunately, due to the low abundance of peptides and poor ionization efficiency^9, 10^, direct analysis still remains a big challenge. Herein, enrichment of glycopeptides and phosphopeptides from the complicated bio-samples before MS analysis is an indispensable step.

Various strategies have been proposed to glycopeptide or phosphopeptide enrichment, including boronic acid affinity chromatography^11, 12^, hydrazine chemistry^13^, hydrophilic interaction chromatography (HILIC)^14, 15^, lectin affinity chromatography^16^, and size exclusion^17^ for glycopeptides, while immobilized metal affinity chromatography (IMAC)^18^, metal oxide affinity chromatography (MOAC)^19^, immunoprecipitation^20^ and ion-exchange chromatography^21^ for phosphopeptides. Among them, HILIC materials, including metal-organic frameworks^22^, monoliths^23^, and nanoparticles^24^, have been regarded as the most potential materials for glycopeptide enrichment with excellent performance, and could be applied to phosphopeptide enrichment based on IMAC techniques, which aroused great interest and popularity due to handy operation, simple enrichment process, low bias to different types of peptides, and mild conditions for MS analysis^25^. However, few people have thought of creating only one material, which could be used for two purposes. (“One for Two”) It brought us to view of finding a novel hydrophilic material, which could be applied to not only glycopeptide enrichment, but also phosphopeptide enrichment, combining with IMAC, that is to say “One for Two”.

In the past decades, metal-organic frameworks (MOFs), a fascinating class of porous materials, have gained much popularity as a tool of separation and enrichment due to its high surface area, adjustable pore size and easy-to-functionalize^26^. MOFs, which are consisted of metal ions and organic ligands, have been employed in separation^27^, gas adsorption, drug delivery^28^, and so on. Recently, metal–organic frameworks have been applied to proteomics, such as, endogenous peptide enrichment^29^, glycopeptide enrichment^30, 31^, enzyme immobilization^32^, etc. For this reason, a new spot hit our head to create a promising material, combining the hydrophilic interaction, IMAC techniques and MOF, further applied to glycoproteome and phosphoproteome analysis. TiO_2_ and Ti (IV)-IMAC materials have been reported before^33^, and it made a very meaningful and attractive work. With only one material, enrichment of both glycopeptides and phosphopeptides could be performed. Inspired by this beautiful research, developing a novel magnetic MOFs to enrich both glycopeptides and phosphopeptides was catchy. As reported, MOFs was endowed with huge surface area, adjustable pore size and easy-to-synthesize and functionalize so it would be meaningful to develop such a material for two purposes.

Herein, to improve the enrichment performance, reduce the trouble in synthesis and cut the time in separation, a facile route was first proposed for preparation of a “One for Two” hydrophilic magnetic amino-functionalized metal-organic framework by modifying UiO-66-NH_2_ (Zr-MOF) onto the polydopamine (PDA)-coated magnetic microspheres (Fe_3_O_4_@PDA@UiO-66-NH_2_). Zr was confirmed that it had strong binding with phosphopeptides and the ligand with amino functioned could be applied to glycopeptide enrichment via hydrophilic interaction. As a core-shell-shell structure, MOFs with 2-aminoterephthalic acid (denoted as H_2_BDC-NH_2_) as the ligand were easily synthesized and with a hydrophilic surface. The as-prepared MOFs exhibited strong magnetic responsiveness and excellent hydrophilicity and strong binding between Zr and phosphopeptides, so a promising future for excellent performance in glycopeptide and phosphopeptide enrichment could be anticipated.

## Results

### Characterization of Fe_3_O_4_@PDA@UiO-66-NH_2_

**Figure 1:**
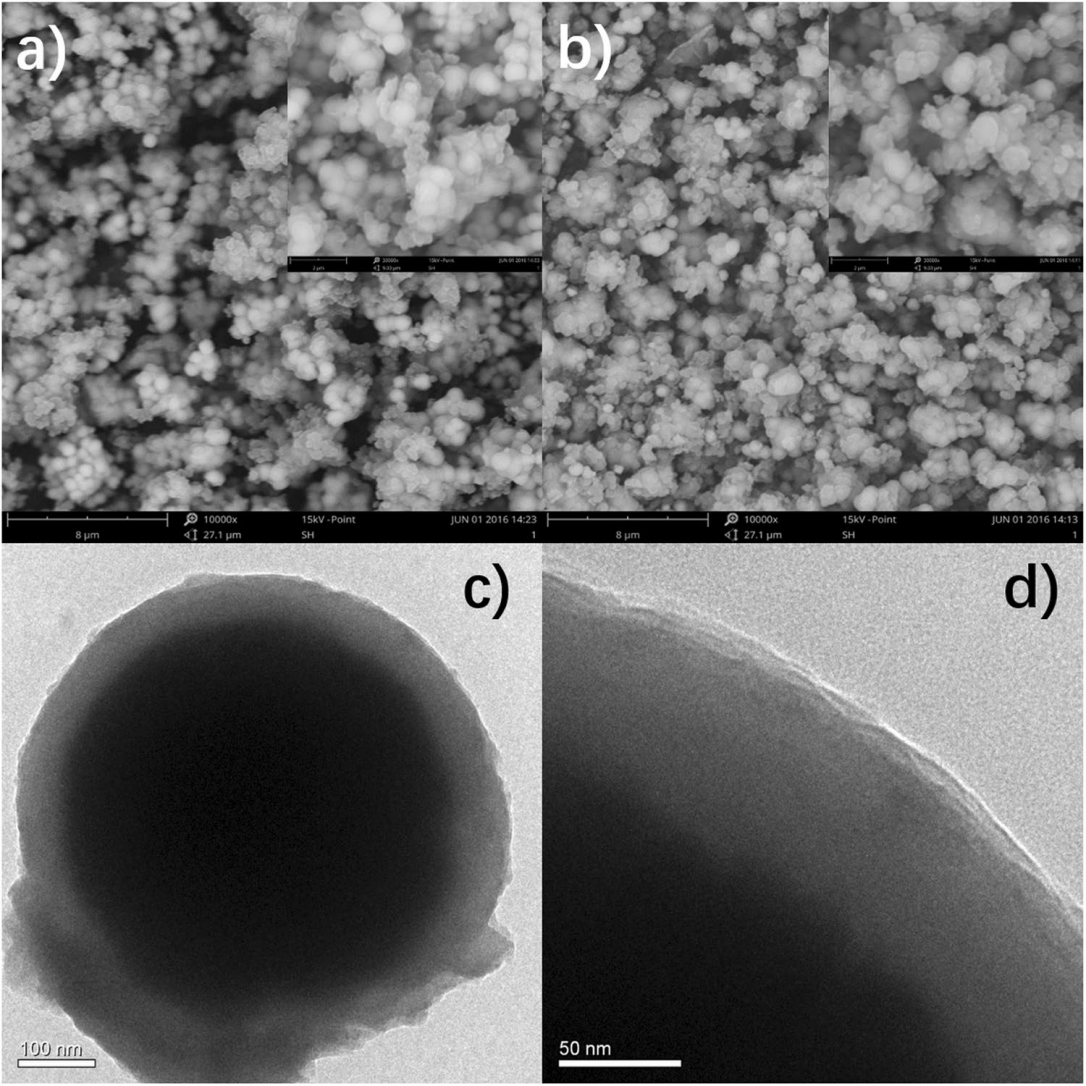
SEM images of (**a**) Fe_3_O_4_@PDA and (**b**) Fe_3_O_4_@PDA@UiO-66-NH_2_; TEM images of (**c**,**d**) Fe_3_O_4_@PDA@UiO-66-NH_2_.

Transmission electron microscopy (TEM) and Scanning electron microscopy (SEM) were used here to confirm the microstructure of Fe_3_O_4_@PDA@UiO-66-NH_2_. Figure 1a shows the SEM images of Fe_3_O_4_@PDA, exhibiting a thin polymer-shell outside the spherical Fe_3_O_4_ microspheres. After modified with UiO-66-NH_2_, the surface of as-prepared MOFs is crystallized and distinct from the smooth surface of Fe_3_O_4_@PDA. EDX analysis of Fe_3_O_4_@PDA@UiO-66-NH_2_ further confirmed the existence of Zr. (Supporting Information Figure S2) TEM image (Fig. 1c) of Fe_3_O_4_@PDA@UiO-66-NH_2_ shows that MOF shell is grafted onto the Fe_3_O_4_@PDA nanoparticles and PDA@MOF shell-shell is around 70 nm with the Fe_3_O_4_ core unchanged. TEM image (Fig. 1d) further exhibits the tiny pore from the magnified MOF shell, which is consistent with the 3.11 nm of the pore size from the BET analysis results. The surface area is 136.36 m^2^/g, which is extremely large based on the rough Fe_3_O_4_ core (Supporting Information Figure S3).

Magnetic responsiveness is crucial for time-saving and simple application in the following enrichment process. Herein, magnetic core is introduced inside the hydrophilic MOFs and magnetic properties of Fe_3_O_4_, Fe_3_O_4_@PDA and Fe_3_O_4_@PDA@UiO-66-NH_2_ were measured via SQUID magnetometer. (Supporting Information Figure S4) As expected, although the core-shell-shell MOFs exhibited decreasing magnetization values when compared to Fe_3_O_4_, it still showed superparamagnetic properties with no coercivity. Apart from magnetic responsiveness, hydrophilicity is another merit of this MOF. After dissolving in DI water, it can maintain as a homogeneous solution for a relatively long time (Supporting Information Figure S5).

FT-IR spectra (Supporting Information Figure S6) and Raman spectra (Supporting Information Figure S7) were recorded for Fe_3_O_4_, Fe_3_O_4_@PDA, and Fe_3_O_4_@PDA@UiO-66-NH_2_ respectively and showed obvious characteristic peaks of Fe-O-Fe vibration (560 cm^−1^) and specific groups, like –COOH (3400 cm^−1^), aromatic rings (1500–1600 cm^−1^ in Raman), etc.

Wide-angle X-ray diffraction patterns of Fe_3_O_4_@PDA@UiO-66-NH_2_ were recorded to confirm the structure of magnetic MOFs (Supporting Information Figure S8). Diffraction peaks at 2θ = 5.2, 7.0, 12.3, 18.2 and 22.3° were from the UiO-66-MOF, while 2θ = 30.3, 35.4, 43.2, 57.2 and 63.0° were from the Fe_3_O_4_ lattice. Zeta potential measurements were operated in ethanol solution (Supporting Information Figure S9). The values dropped first and then went up, which was ascribed to the negative acidic catechol hydroxyl groups and MOFs modification.

### Enrichment of glycopeptides and phosphopeptides from standard proteins using Fe_3_O_4_@PDA@UiO-66-NH_2_

**Figure 2:**
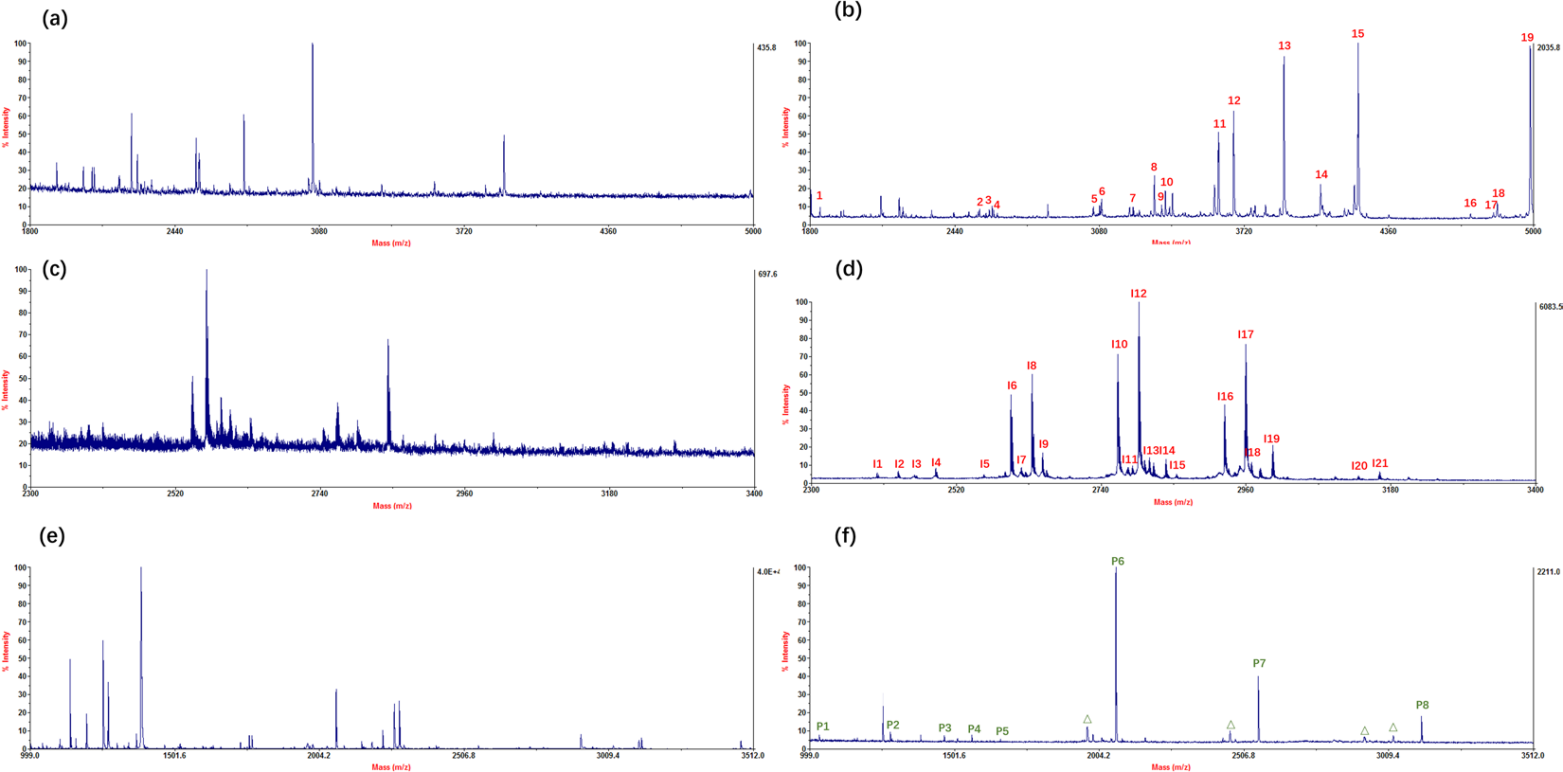
MALDI-TOF mass spectra for the glycopeptide enrichment from 250 fmol/μL HRP tryptic digest: (**a**) before enrichment, and (**b**) after treatment; from 1 pmol/μL IgG: (**c**) before enrichment, and (**d**) after treatment; from 200 fmol/μL β-Casein: (**e**) before enrichment, and (**f**) after treatment, where glycopeptides were marked with Arabic Numerals in red, phosphopeptides were marked with Arabic Numerals in green, and Δ indicates the losses of phosphoric acid.

The workflow of glycopeptide or phosphopeptide enrichment is illustrated in Figure S1. The ability of the as-prepared hydrophilic magnetic MOFs for glycopeptide enrichment was investigated by using HRP digests and human IgG digests. As shown in Fig. 2a, before enrichment, the signals of N-glycopeptides were overwhelmed by the non-glycopeptides. Notably, after selective enrichment, the intensity of glycopeptides was significantly increased and 19 glycopeptides could be observed, anticipating an excellent enrichment performance. What’s more, enrichment of IgG digests also exhibited excellent performance with 21 glycopeptides identified. (Figure 2c,d) Detailed information of glycopeptide of HRP and IgG digests could be found in the Supporting Information Table S1 and Table S2. The ability of the as-prepared hydrophilic magnetic MOFs for phosphopeptide enrichment was investigated by using β-Casein digest. As shown in Fig. 2e,f, after enrichment, the peaks of phosphopeptides appeared with high intensities and a clear background. Detailed information of phosphopeptide of β-Casein digests could be found in the Supporting Information Table S3.

HRP (β-Casein) was chosen as the standard glycoprotein (phosphoprotein) to prove the properties of Fe_3_O_4_@PDA@UiO-66-NH_2_ in the following analysis. The reusability (Supporting Information Figure S10) and stability (Supporting Information Figure S11) of the hydrophilic magnetic MOFs were also investigated. The spectrum of 250 fmol/μL HRP tryptic digest and 200 fmol/μL β-Casein using the five-times-recycled MOFs and the MOFs stored at −20 °C for a month showed almost the same enrichment performance as that using the first-time freshly-prepared MOFs, exhibiting the excellent reusability and stability.

**Figure 3:**
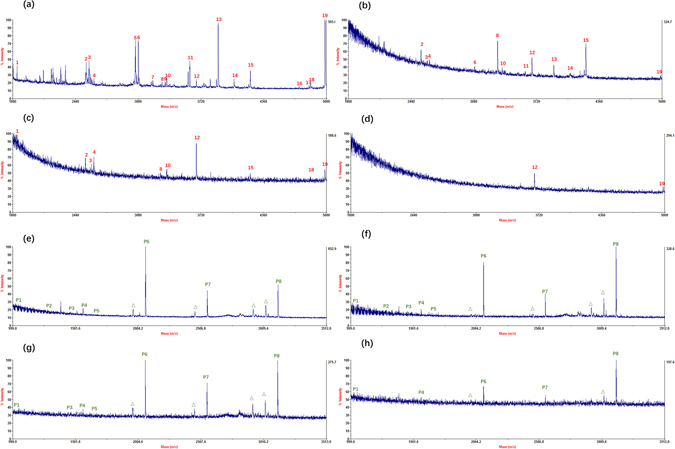
MALDI-TOF mass spectra for the glycopeptide enrichment from HRP tryptic digest: (**a**) 25 fmol/μL, (**b**) 5 fmol/μL, (**c**) 1 fmol/μL, and (**d**) 0.2 fmol/μL; for the phosphopeptide enrichment from β-Casein tryptic digest: (**e**) 25 fmol/μL, (**f**) 1 fmol/μL, (**g**) 0.2 fmol/μL, and (**h**) 0.02 fmol/μL, where glycopeptides were marked with Arabic Numerals in red, phosphopeptides were marked with Arabic Numerals in green, and Δ indicates the losses of phosphoric acid.

To further confirm the excellent capacity and sensitivity of the MOFs, different concentrations of HRP digests and β-Casein digests were applied to the MOFs. As shown in Fig. 3, with the decreasing concentration (25, 5, 1, and 0.2 fmol/μL) of HRP and (25, 1, 0.2, 0.02 fmol/μL) of β-Casein, several glycopeptides or phosphopeptides could still be identified after enrichment. Even though the concentration of HRP was as low as 0.2 fmol/μL, two characteristic peaks of glycopeptides dominated the spectrum (Fig. 3d), exhibiting a relatively lower detection limit while compared to previous reports^30^. Through the different concentrations from high to ultra-low, by calculation, the maximum capacity of MOFs towards HRP (glycoproteins) was 4 mg/g, down to 0.8 μg/g; while the maximum capacity of MOFs towards β-Casein (phosphoproteins) was 0.8 mg/g, down to 0.08 μg/g.

**Figure 4:**
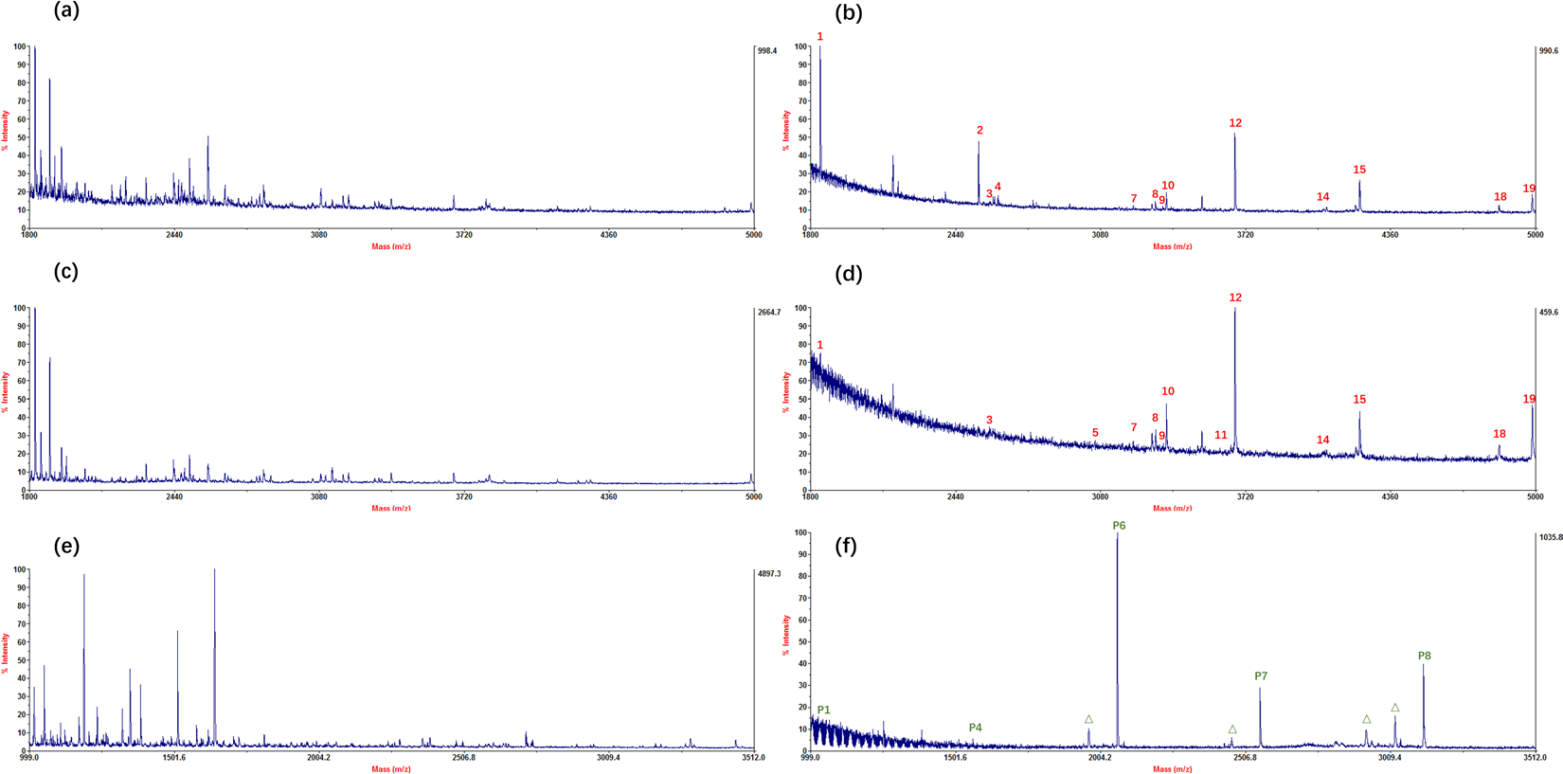
MALDI-TOF mass spectra for the glycopeptide enrichment from a mixture of HRP and BSA at a mass ratio of 1:50: (**a**) before enrichment, and (**b**) after enrichment; 1:100: (**c**) before enrichment, and (**d**) after enrichment; for the phosphopeptide enrichment from a mixture of β-Casein and BSA at a mass ratio of 1:500: (**e**) before enrichment, and (**f**) after enrichment, where glycopeptides were marked with Arabic Numerals in red, phosphopeptides were marked with Arabic Numerals in green, and Δ indicates the losses of phosphoric acid.

The selectivity of Fe_3_O_4_@PDA@UiO-66-NH_2_ toward glycopeptides and phosphopeptides was investigated via the mixture of HRP (β-Casein) and BSA tryptic digests. With a mass ratio of 1:50 (HRP:BSA), as shown in Fig. 4a, glycopeptides could hardly be identified after direct analysis. However, after enrichment, most glycopeptides dominated the spectra with a relatively high signal intensity. With an increasing ratio as 1:100, although the absolute intensity decreased, the relative intensity of glycopeptides showed the high selectivity of the MOFs for capturing glycopeptides. For phosphopeptides, the ratio could be as high as 1:500 (β-Casein:BSA) with outstanding performance. Due to the instability of MALDI TOF 5800, all the experiments should be done at the same time in theory. However, it was unrealistic so all the experiments were performed under same experimental conditions. The recovery was confirmed here and almost no non-glycopeptides appeared after enrichment. (Supporting Information Figure S12) What’s more, the recovery of the MOFs was further confirmed through a parallel test, around 94% (Supporting Information Figure S13).

### Enrichment of glycopeptides and phosphopeptides from human serum using Fe_3_O_4_@PDA@UiO-66-NH_2_

**Figure 5:**
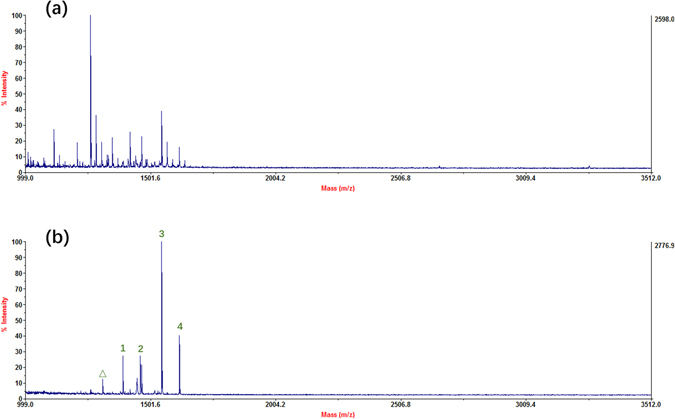
MALDI-TOF mass spectra for the phosphopeptide enrichment from human serum: (**a**) before enrichment, and (**b**) after enrichment, where phosphopeptides were marked with Arabic Numerals in green, and Δ indicates the losses of phosphoric acid.

Inspired by the excellent enrichment performance above, the as-prepared MOFs were applied to the enrichment of glycopeptides and phosphopeptides from human serum digest and analyzed by LC-MS/MS and MALDI respectively. According to the procedure, the eluted glycopeptides were deglycosylated by PNGase F and analyzed by Nano-HPLC-MS/MS. The human Uniprot-SwissProt database (release 2015_03_11, with 20199 entries) was chosen as a database, and a total of 307 N-glycosylation peptides from 121 different glycoproteins were identified, showing excellent and promising potential in glycoproteome research. Detailed information could be found in Supporting Information Table S4. For phosphopeptides, four endogeneous phosphopeptides and one peak of loss of phosphoric acid could be identified from the spectrum (Fig. 5) and the detailed information of phosphopeptides from human serum was listed in Supporting Information Table S5. From the human serum digests, a total of 33 phosphopeptides from 16 different phosphoproteins were identified, apart from the endogenous phosphopeptide enrichment from human serum without additional pretreatment, it became a breakthrough of phosphopeptide enrichment from human serum digests. Detailed information could be found in Supporting Information Table S6.

## Discussion

To dates, Mass spectrometry (MS) has become one of the most common tools to identify protein glycosylation because of its high throughput, fast speed and high sensitivity. Unfortunately, due to the low abundance of glycopeptides and phosphopeptides and poor ionization efficiency, direct analysis of certain peptides still remains a big challenge. Herein, enrichment of glycopeptides and phosphopeptides from the complicated bio-samples before MS analysis is an indispensable step.

A new spot hit our head to create a promising hydrophilic MOF material, which could be applied to not only glycopeptide enrichment, but also phosphopeptide enrichment, combining with IMAC, that is to say “One for Two”.

Herein, a facile route was first proposed for preparation of a “One for Two” hydrophilic magnetic amino-functionalized metal-organic framework by modifying UiO-66-NH_2_ (Zr-MOF) onto the polydopamine (PDA)-coated magnetic microspheres (Fe_3_O_4_@PDA@UiO-66-NH_2_). Based on hydrophilic interaction, it was applied to glycopeptide enrichment, while based on strong binding between Zr and phosphopeptides, it was applied to phosphopeptide enrichment. Fe_3_O_4_ (strong magnetic responsiveness), PDA (excellent biological amphiphilicity) and MOFs (high surface area) are combined here so the as-prepared material is deemed to enrich glycopeptides and phosphopeptides with high sensitivity and selectivity.

A magnetic core Fe_3_O_4_ was easily prepared, followed by self- polymerization of dopamine, creating a polydopamine (PDA) onto Fe_3_O_4_. Thanks to the hydroxyl and amino group of PDA, Zr^3+^ was easily adhered to the surface, inducing the following one-pot MOF reaction with amino ligand. Owing to its large surface area, strong magnetic responsiveness, high selectivity and sensitivity, it showed great potential in glycoproteome and phosphoproteome research, exhibiting excellent performance with high sensitivity (0.2 fmol/μL) and selectivity (1:100) for glycopeptide, while 0.02 fmol/μL and 1:500 for phosphopeptide. In addition, the novel as-prepared MOFs was successfully applied to complex biological samples, like human serum (with 307 N-glycosylation peptides from 121 different glycoproteins, 33 phosphopeptides from 16 different phosphoproteins and four endogenous phosphopeptides identified), revealing its promising application in glycopeptide and phosphopeptide enrichment.

## Methods

### Synthesis of Fe_3_O_4_@PDA@UiO-66-NH_2_

**Figure 6:**
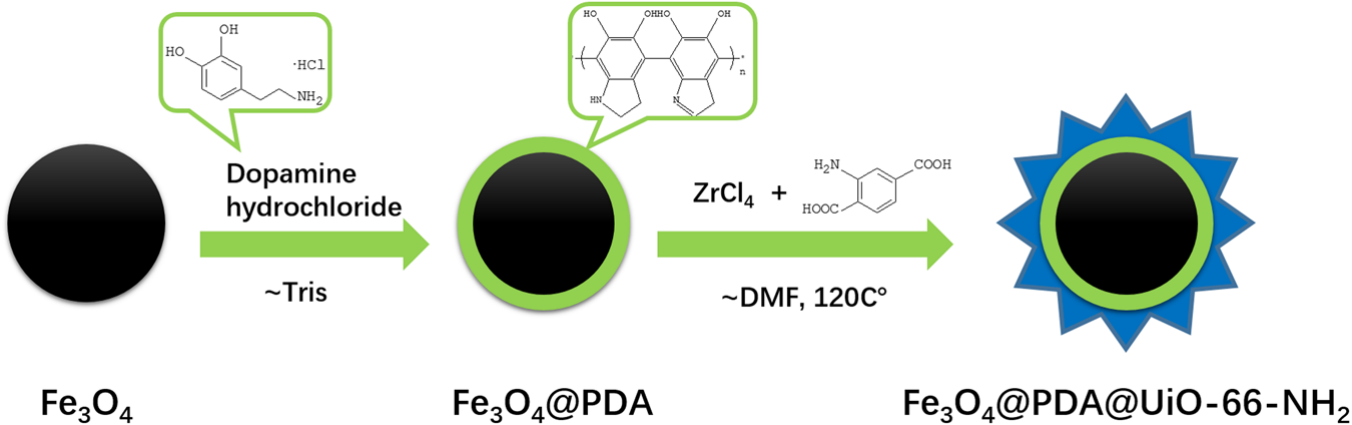
The Synthetic Route for Fe_3_O_4_@PDA@UiO-66-NH_2_.

A facile route for Fe_3_O_4_@PDA@UiO-66-NH_2_ is proposed in Fig. 6. The magnetic microspheres were prepared via a well-known solvo-thermal reaction^34^. Then, the magnetic particles were coated with a PDA layer through the polymerization of dopamine in basic atmosphere. The obtained PDA coated magnetic particles (denoted as Fe_3_O_4_@PDA) were dispersed in N,N-dimethylformamide (DMF), containing ZrCl_4_ and 2-aminoterephthalic acid as MOFs precursors. Then the mixed solution was heated under 120 °C for 45 min. Finally, Fe_3_O_4_@PDA@UiO-66-NH_2_ was successfully prepared by this simple one-pot reaction.

### Characterization and Measurements

Scanning electron microscopy (SEM), energy dispersive X-ray (EDX), transmission electron microscopy (TEM), fourier transform infrared spectra (FT-IR), raman spectra (Raman), magnetization measurement, powder X-ray diffraction patterns (XRD), magnetization measurements and nitrogen sorption isotherms and zeta potential measurements were measured and detailed information were illustrated in SI.

### Enrichment of glycopeptides from standard glycoproteins and human serum

In brief, 200 μg of Fe_3_O_4_@PDA@UiO-66-NH_2_ was added into 100 μL standard peptide mixture and the solution was incubated at 37 °C for 30 min. 90%ACN/1%TFA was chosen as the loading buffer here to establish a hydrophobic environment. After washing by the loading buffer and 80%ACN/1%H_3_PO_4_ to remove the non-glycopeptides^30^, the captured glycopeptides were eluted by 30%ACN/0/1%FA at 37 °C for 20 min and then analyzed by matrix-assisted laser desorption ionization time-of-flight mass spectrometry (MALDI-TOF MS). For human serum, after the same enrichment and elution process, the elution was lyophilized first and then redissolved in 25 mM NH_4_HCO_3_ solution and incubated with 1 μL PNGase F at 37 °C overnight. After lyophilization, it was transferred to nano high performance liquid chromatography- mass spectrometry (Nano-HPLC-MS/MS) analysis. The protocol of LC-MS/MS analysis could be found in Supporting Information in detail.

### Enrichment of phosphopeptides from standard phosphoproteins and human serum

The enrichment steps are the same as glycopeptide enrichment except for the loading and washing buffer were replaced by 50%ACN/0.1%TFA and the elution was replaced by 0.4 M NH_3_·H_2_O. For human serum, after the same enrichment and elution process, the elution was lyophilized first and then transferred to nano high performance liquid chromatography- mass spectrometry (Nano-HPLC-MS/MS) analysis.

## Electronic supplementary material

Designed synthesis of a “One for Two” hydrophilic magnetic amino-functionalized metal-organic framework for highly efficient enrichment of glycopeptides and phosphopeptides
