# Supplementary material for: Designed synthesis of a “One for Two” hydrophilic magnetic amino-functionalized metal-organic framework for highly efficient enrichment of glycopeptides and phosphopeptides
Source: Sci Rep. 2017 Apr 25;7:1162. doi: 10.1038/s41598-017-01341-y (PMC5430903; doi:10.1038/s41598-017-01341-y)
Supplement: Supplementary file 1 — Designed synthesis of a “One for Two” hydrophilic magnetic amino-functionalized metal-organic framework for highly efficient enrichment of glycopeptides and phosphopeptides [file 41598_2017_1341_MOESM1_ESM.pdf]

Designed synthesis of a "One for Two" hydrophilic magnetic amino-functionalized metal-organic framework for highly efficient enrichment of glycopeptides and phosphopeptides

Supporting Information

Yiqin Xie[1], Chunhui Deng[1,*]

[1]Department of Chemistry and Institutes of Biomedical Sciences, Collaborative Innovation Center of Genetics and Development, Fudan University, Shanghai 200433, China

*Correspondence to chdeng@fudan.edu.cn

Experimental Section

1. Materials and reagents

Zircomiun tetrachloride ($ZrCl_4$), 2-aminoterephthalic acid ($H_2BDC-NH_2$), Phosphoric acid (H_3PO_4), N,N'- Dimethylformamide (DMF), Horseradish peroxidase (HRP), Immunoglobulin G (IgG), β-casein from bovine milk, Albumin from bovine serum (BSA), Trypsin from bovine pancreas, Formic Acid (FA) and Trifluoroacetic acid (TFA) were purchased from Sigma-Aldrich. Iron (III) chloride hexahydrate ($FeCl_3$•$6H_2O$), Sodium acetate (CH_3COONa), Ethanol, Ethylene glycol, Ammonium hydroxide (NH3•H2O) and Ammonium bicarbonate (NH_4HCO_3) were purchased from Sinopharm Chemical Reagent Co., Ltd. Acetonitrile (ACN) was purchased from Merck. Tris(hydroxymethyl) aminomethane (Tris), and 2,5-dihydroxybenzoic acid (DHB) were purchased from J&K Scientific. Human serum was supplied by Zhongshan Hospital. All other chemicals are of the top grade commercially available. Distilled water was purified using Milli-Q system (Millipore, Bedford, MA).

2. Synthesis of Fe_3O_4@PDA@UiO-66-NH_2

Fe_3O_4 nanoparticles were prepared via a well-known solvo-thermal reaction.[1] In detail, 1.35 g $FeCl_3$•$6H_2O$ was dispersed in 75 mL ethylene glycol under stirring until completely dissolved. Then, 3.60 g smashed CH_3COONa was added into the above solution and under stirring for 30 min until a homogeneous solution was obtained. After that, the solution was transferred into a Teflon-lined stainless-steel autoclave and kept at 200C° for 16h. The obtained Fe_3O_4 was collected by magnetic separation, washed with deionized water and ethanol for several times and finally kept in vacuum oven at 50C°.

Fe_3O_4@PDA was prepared via the polymerization of dopamine in basic solution. In detail, 0.12 g Fe_3O_4 was added into 80 mL Tris buffer, which contained 0.05 g Tris and 0.32 g dopamine hydrochloride. The solution was kept vigorously stirring for 16 h at room temperature. The obtained Fe_3O_4@PDA was collected by magnetic separation, washed with deionized water and ethanol for several times and kept in vacuum oven at 50C°.

Fe_3O_4@PDA@UiO-66-NH_2 was prepared by a simple one-pot reaction. 0.10 g Fe_3O_4@PDA was dispersed in 75 mL DMF of MOFs precursors, containing 0.16 g $ZrCl_4$ (9mM) and 0.12 g (9mM) 2-aminoterephthalic acid (H_2BDC-NH_2) under 120C° for 45 min. The obtained Fe_3O_4@PDA@ UiO-66-NH_2 was collected by magnetic separation, washed with DMF and ethanol for several times and kept in vacuum oven at 50C°.

3. Characterization and measurements

Scanning electron microscopy (SEM) images and Energy dispersive X-ray (EDX) were operated on a Philips XL30 electron microscope (Netherlands) at 20 kV. Transmission electron microscopy (TEM) images were operated on a JEOL 2011 microscope (Japan) at 200 kV. Fourier transform infrared spectra (FT-IR) was operated on a Nicolet Fourier spectrophotometer (U.S.A.). Raman spectra (Raman) was operated on a LabRam-1B Raman spectrometer. Magnetization measurement was operated on a MPMS (SQUID) VSM (U.S.A.). Powder X-ray diffraction patterns (XRD) were operated on a Bruker D4 X-ray diffractometer at 40 kV, 40 mA. Nitrogen sorption isotherms were operated on a Micromeritcs Tristar 3000 analyzer (U.S.A.) at 77 K. Zeta potential measurements were operated on a Nano ZS90 zeta analyzer (Malvern Instruments Ltd.).

4. Sample preparation

The as-prepared Fe_3O_4@PDA@UiO-66-NH_2 was suspended in deionized water to make a dispersion of 10 mg/mL solution for further use.

For glycopeptide: 30 mg DHB was added into 1 mL 50%ACN to make a dispersion of 30 mg/mL solution for further use.

For phosphopeptide: 20 mg DHB was added into 1 mL 50%ACN (containing 1% H_3PO_4) to make a dispersion of 20 mg/mL solution for further use.

Horseradish peroxidase (HRP), Immunoglobulin G (IgG), β-Casein and Albumin from bovine serum (BSA) were dissolved in 25 mM NH_4HCO_3 buffer (pH=8.3), denatured at 100 C° for 10 min and incubated with trypsin (trypsin: protein is 1:50, w/w) at 37 C° for 16 h. The final concentration of HRP was 2 mg/mL, IgG was 4 mg/mL, β-Casein was 2.5 mg/mL and BSA was 5 mg/mL. The obtained tryptic digests were diluted with loading buffer (90%ACN/1%TFA for glycopeptide and 50%ACN/0.1%TFA for phosphopeptide) for further enrichment and analysis.

For glycopeptide enrichment: Human serum (2 μL) was diluted in 198 μL NH_4HCO_3 buffer and denatured for 10 min. The mixture was reduced by 10 mM DTT at 60 C° for 30 min and alkylated by 20 mM IAA at 37 C° for 1 h in the dark. Then the obtained mixture was incubated with trypsin (trypsin: protein is 1:50, w/w) at 37 C° for 16 h. The obtained tryptic digests were lyophilized for further enrichment and analysis.

For phosphopeptide enrichment: Human serum (2 μL) was diluted in 50%ACN/0.1%TFA by 10 fold without additional process.

5. Enrichment of glycopeptides or phosphopeptides from standard tryptic digests

The workflow of glycopeptide or phosphopeptide enrichment is illustrated in Scheme S1.

For glycopeptide enrichment: Detailedly, 200 μg Fe_3O_4@PDA@UiO-66-NH_2 was added into 100 μL mixture of peptides (diluted by 90%ACN/1%TFA) and the obtained solution was vibrated in a vortex at 37 C° for 30 min. After removal of the supernatant under magnetic separation, the MOFs was rinsed with 90%ACN/1%TFA once and 80%ACN/1%H_3PO_4 twice to

remove non-glycopeptides. After that, 5 µL 30%ACN/0.1%FA was added into the MOFs and vibrated in the same vortex to elute the glycopeptides at 37 C° for 20 min. The elution was collected under magnetic separation.

For phosphopeptide enrichment: Detailedly, 200 µg Fe_3O_4@PDA@UiO-66-NH_2 was added into 100 µL mixture of peptides (diluted by 50%ACN/0.1%TFA) and the obtained solution was vibrated in a vortex at 37 C° for 30 min. After removal of the supernatant under magnetic separation, the MOFs was rinsed with 50%ACN/0.1%TFA three times to remove non-phosphopeptides. After that, 10 µL 0.4 M $NH_3 \cdot H_2O$ was added into the MOFs and vibrated in the same vortex to elute the phosphopeptides at 37 C° for 20 min. The elution was collected under magnetic separation.

Finally, 1 µL of elution was deposited on a MALDI plate and let dry, followed by 0.5 µL DHB and let dry and analyzed by matrix-assisted laser desorption ionization time-of-flight mass spectrometry (MALDI-TOF MS).

6. MALDI-TOF MS analysis

MALDI-TOF MS measurements were operated on a 5800 Proteomics Analyzer (Applied Biosystems, U.S.A.) in positive ion mode at the Nd:YAG laser of 355 nm, the repetition rate of 200 Hz and the acceleration voltage of 20 kV.

7. Enrichment of glycopeptides or phosphopeptides from tryptic digest mixtures of HRP/β-Casein and BSA

200 µg Fe_3O_4@PDA@UiO-66-NH_2 was added into 100 µL tryptic digest mixtures of HRP/β-Casein and BSA at a given mass ratio. The enrichment process was the same as the section 5. The collected elution was analyzed by MALDI-TOF MS.

8. Enrichment of glycopeptides or phosphopeptides from healthy human serum

For glycopeptide enrichment: The lyophilized tryptic digests of human serum were redissolved in 100 µL loading buffer and then 400 µg Fe_3O_4@PDA@UiO-66-NH_2 was added into the above solution. The enrichment process was the same as the section 5. The collected elution was lyophilized and redissolved in 50 µL 25 mM NH_4HCO_3 solution, and then 1 µL PNGase F was added at 37C° for 16 h to remove the glycans. The collected solution was lyophilized again for further LC-MS/MS analysis.

For phosphopeptide enrichment:

For human serum without treatment: It was the same as the standard phosphoprotein. Detailedly, 200 µg Fe_3O_4@PDA@UiO-66-NH_2 was added into 200 µL serum dilution, containing 10 µL 10-fold-diluted human serum and 190 µL loading buffer. After enrichment, washing and elution, 1 µL of elution was deposited on a MALDI plate and let dry, followed by 0.5 µL DHB and let dry and analyzed by matrix-assisted laser desorption ionization time-of-flight mass spectrometry (MALDI-TOF MS).

For human serum digests: The lyophilized tryptic digests of human serum were redissolved in 100 µL loading buffer and then 400 µg Fe_3O_4@PDA@UiO-66-NH_2 was added into the above solution. The enrichment process was the same as the section 5. The collected elution was lyophilized for further LC-MS/MS analysis.

9. Nano High Performance Liquid Chromatography- Mass Spectrometry (LC-MS/MS) analysis of glycopeptides and phosphopeptides

The peptide samples were resuspended with 10 µL solvent A respectively (A: water with 0.1% formic acid; B: ACN with 0.1% formic acid), separated by nanoLC and analyzed by

on-line electrospray tandem mass spectrometry. The experiments were performed on an EASY-nLC 1000 system (Thermo Fisher Scientific, Waltham, MA) connected to an Orbitrap Fusion mass spectrometer (Thermo Fisher Scientific, San Jose, CA) equipped with an online nano-electrospray ion source. 4 µL peptide sample was loaded onto the analytical column (Acclaim PepMap C18, 75 µm x 50 cm) with a linear gradient, from 2% B to 40% B in 110 min. The column was re-equilibrated at initial conditions for 10 min. The column flow rate was maintained at 200nL/min. The electrospray voltage of 2.0 kV versus the inlet of the mass spectrometer was used.

The Orbitrap Fusion mass spectrometer was operated in the data-dependent mode to switch automatically between MS and MS/MS acquisition. Survey full-scan MS spectra (m/z 350-1500) were acquired in Orbitrap with a mass resolution of 120 000 at m/z 200. The AGC taget was set to 300 000, and the maximum injection time was 50ms. MS/MS acquisition was performed in Orbitrap with 3 s cycle time, the resolution was 30 000 at m/z 200. The intensity threshold was 50 000, and the maximum injection time was 150 ms. The AGC target was set to 200 000, and the isolation window was 2 m/z. Ions with charge states 2+, 3+, and 4+ were sequentially fragmented by higher energy collisional dissociation (HCD) with a normalized collision energy (NCE) of 30%, fixed first mass was set at 110. In all cases, one microscan was recorded using dynamic exclusion of 50 seconds.

10. Database Search

For glycopeptide search:

Tandem mass spectra were extracted by Proteome Discoverer software (Thermo Fisher Scientific, version 1.4.0.288). Charge state deconvolution and deisotoping were not performed. All MS/MS samples were analyzed using Mascot (Matrix Science, London, UK; version 2.3). Mascot was set up to search the human Uniprot-SwissProt database (release 2015_03_11, with 20199 entries) assuming the digestion enzyme trypsin. Mascot was searched with a fragment ion mass tolerance of 0.050 Da and a parent ion tolerance of 10.0 PPM. Carbamidomethyl of cysteine was specified in Mascot as fixed modifications. Oxidation of methionine and deamidation of asparagine were specified in Mascot as a variable modification. Percolator algorithm was used to control peptide level false discovery rates (FDR) lower than 1%.

Only the identified deamination sites which were confirmed to the N-glycosylation consensus sequence (n-!P-[S/T]) were considered as glycosylation sites.

For phosphopeptide search:

The raw mass spectrometry data files were extracted using the Proteome Discoverer software (Thermo Fisher Scientific, version 1.4.0.288), and the MS/MS results were analyzed using Mascot (Matrix Science, London, UK; version 2.3). The search parameters were as follows: Database was specified in Mascot as Uniprot-SwissProt (Taxonomy: human, 20199 entries), enzyme was specified as trypsin, a fragment ion mass tolerance was specified as 0.050 Da and a parent ion tolerance was specified as 10.0 PPM. Carbamidomethyl of cysteine was specified in Mascot as fixed modifications. Variable modifications of +80 Da for Ser, Thr, and Tyr, and +16 Da for Met. Peptide level false discovery rates (FDR) were controlled lower than 1% by the percolator algorithm. Using the PhosphoRS 3.0 software to calculate the probability of phosphorylation site.

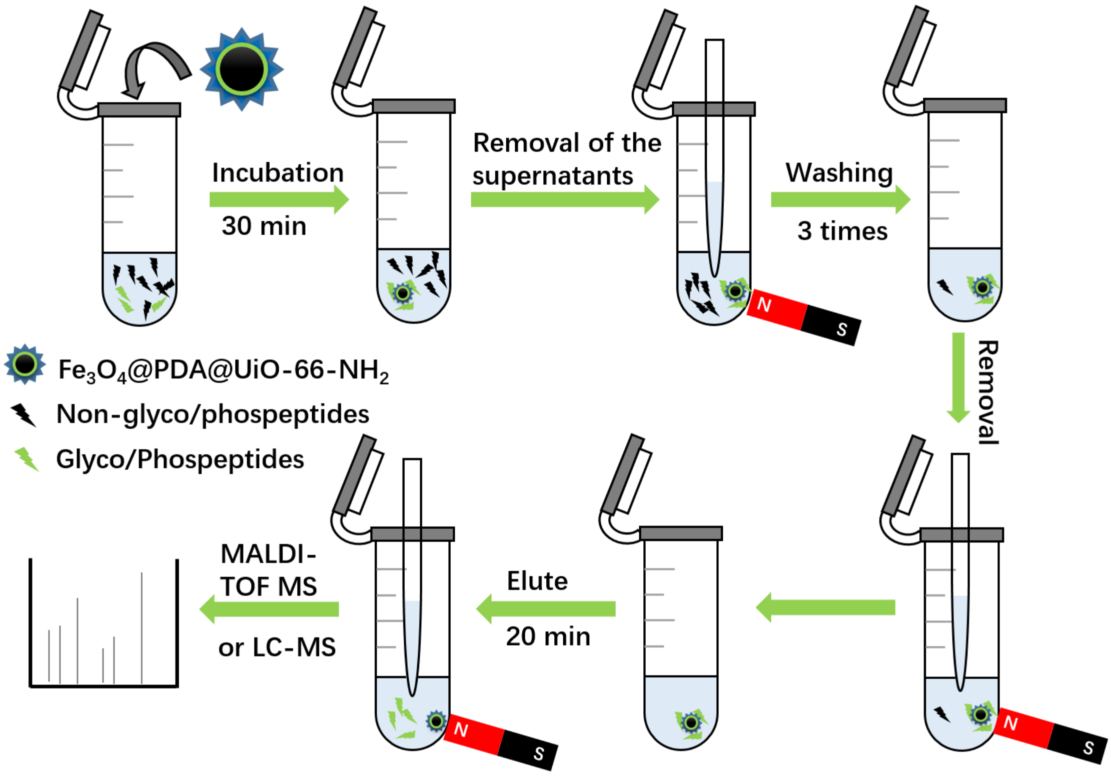

Figure S1. Workflow of glycopeptide or phosphopeptide enrichment from biological samples using Fe_3O_4@PDA@UiO-66-NH_2.

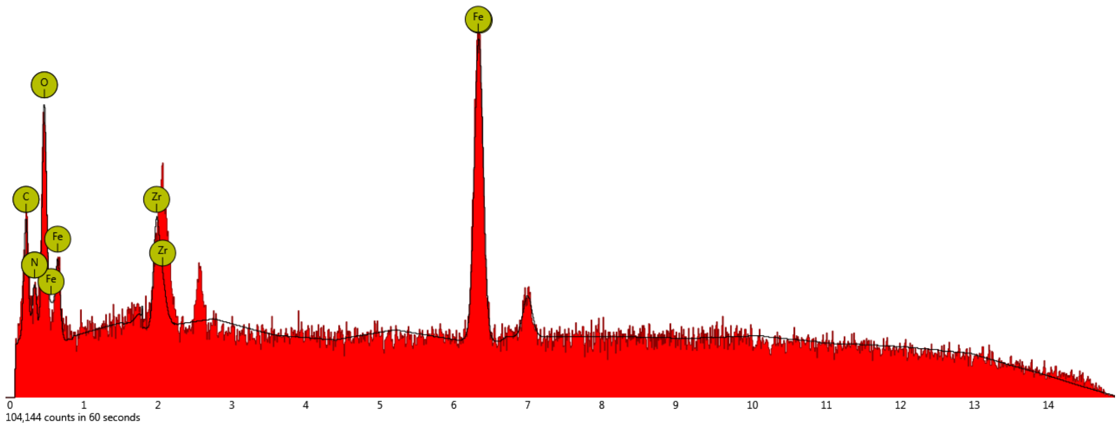

Element Number	Element Symbol	Element Name	Weight Concentration	Error
26	Fe	Iron	41.9	0.1
8	O	Oxygen	20.0	0.2
40	Zr	Zirconium	8.0	1.1
6	C	Carbon	18.2	1.0
7	N	Nitrogen	11.9	0.8

Figure S2. Energy dispersive X-ray (EDX) spectrum data of Fe_3O_4@PDA@UiO-66-NH_2.

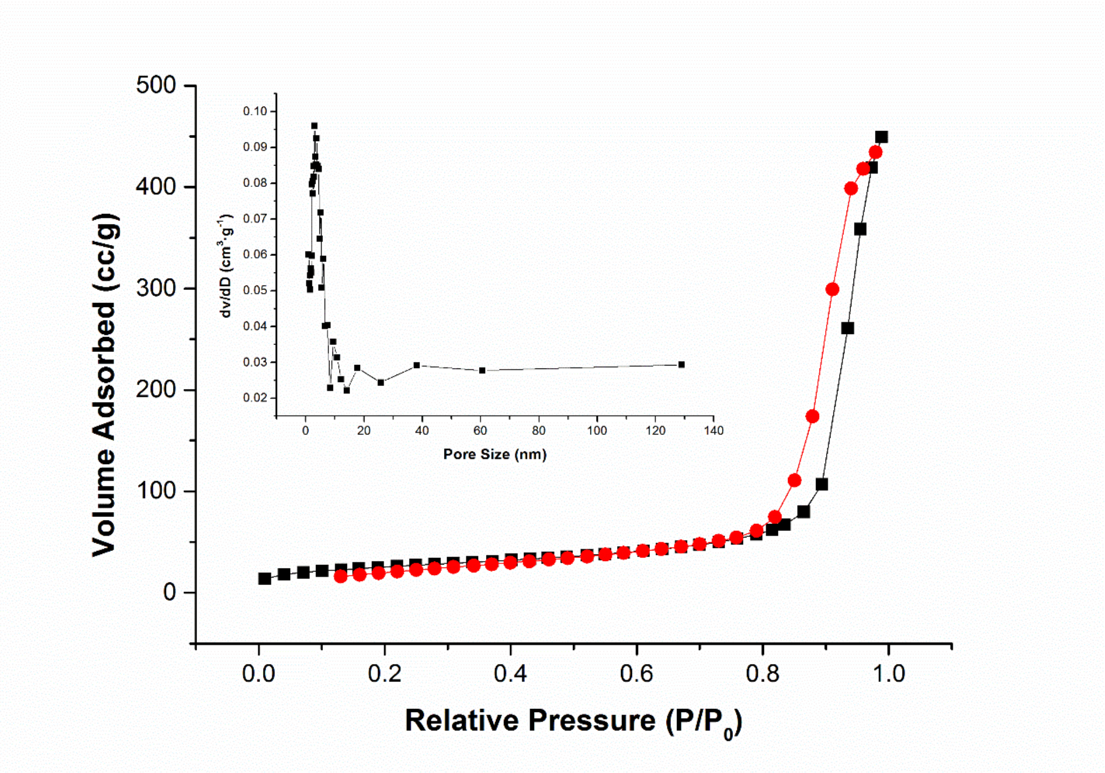

Figure S3. Nitrogen sorption isotherms and pore size distribution of Fe_3O_4@PDA@UiO-66-NH_2.

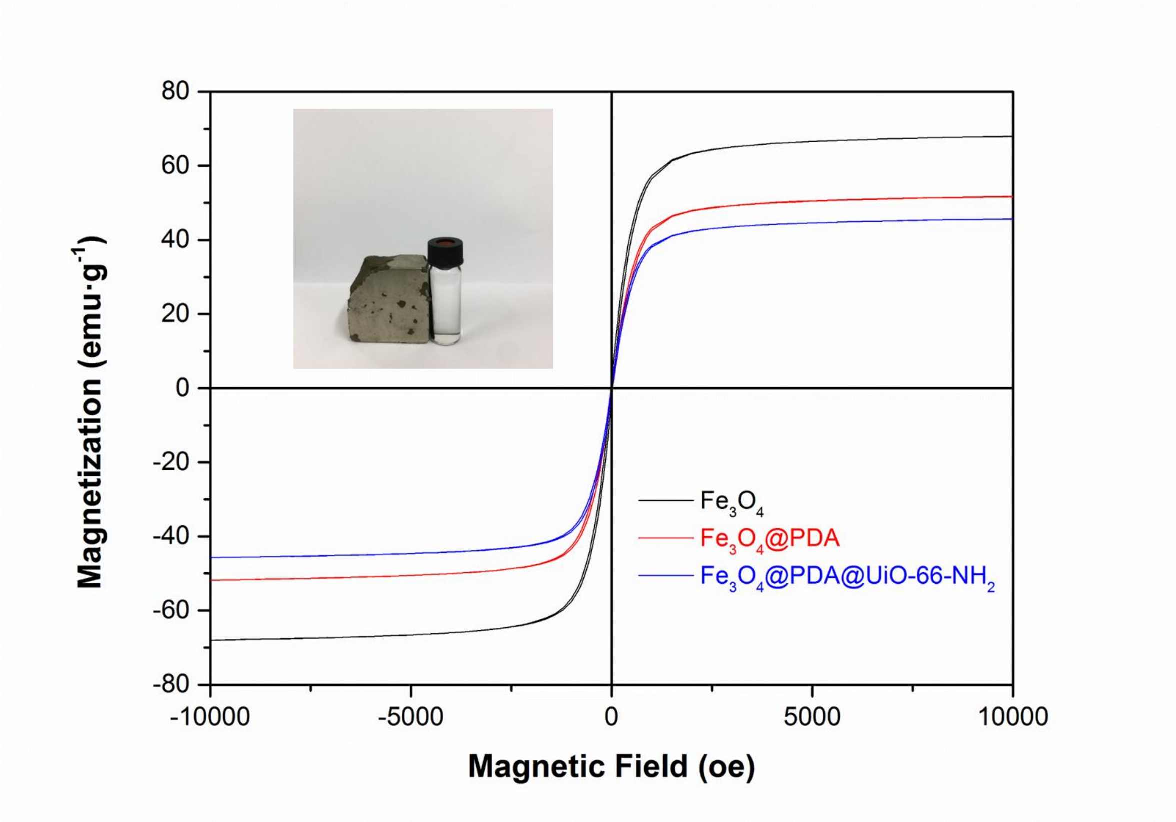

Figure S4. Magnetic hysteresis curves of Fe_3O_4, Fe_3O_4@PDA and Fe_3O_4@PDA@UiO-66-NH_2. The saturation magnetization values of Fe_3O_4, Fe_3O_4@PDA and Fe_3O_4@PDA@UiO-66-NH_2

were 67.9 emu·g^{-1}, 51.6 emu·g^{-1} and 45.6 emu·g^{-1} respectively.

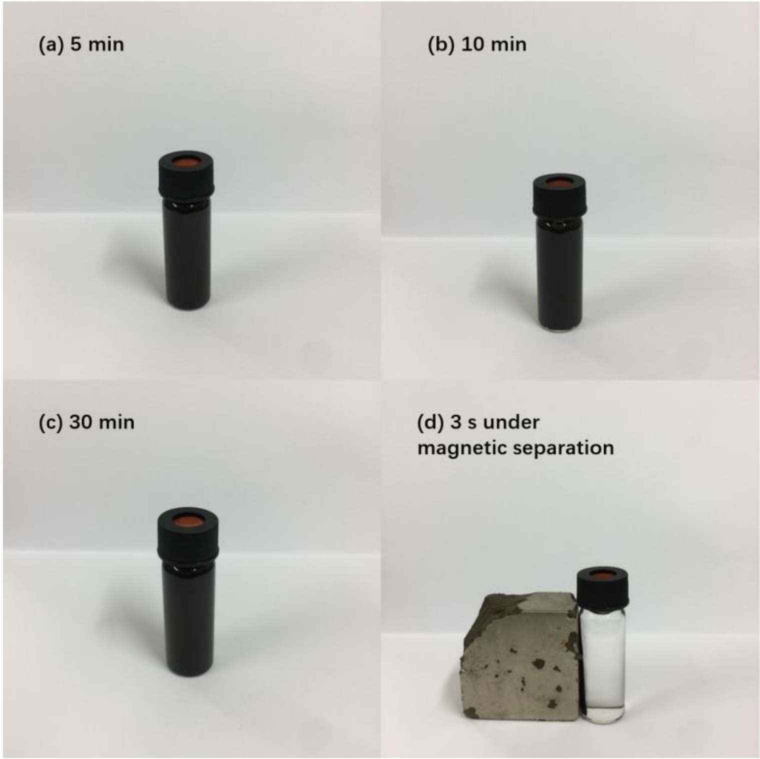

Figure S5. MOFs dispersion in water solution: (a) 5 min, (b) 10 min, (c) 30 min, and (d) 3 s under magnetic separation.

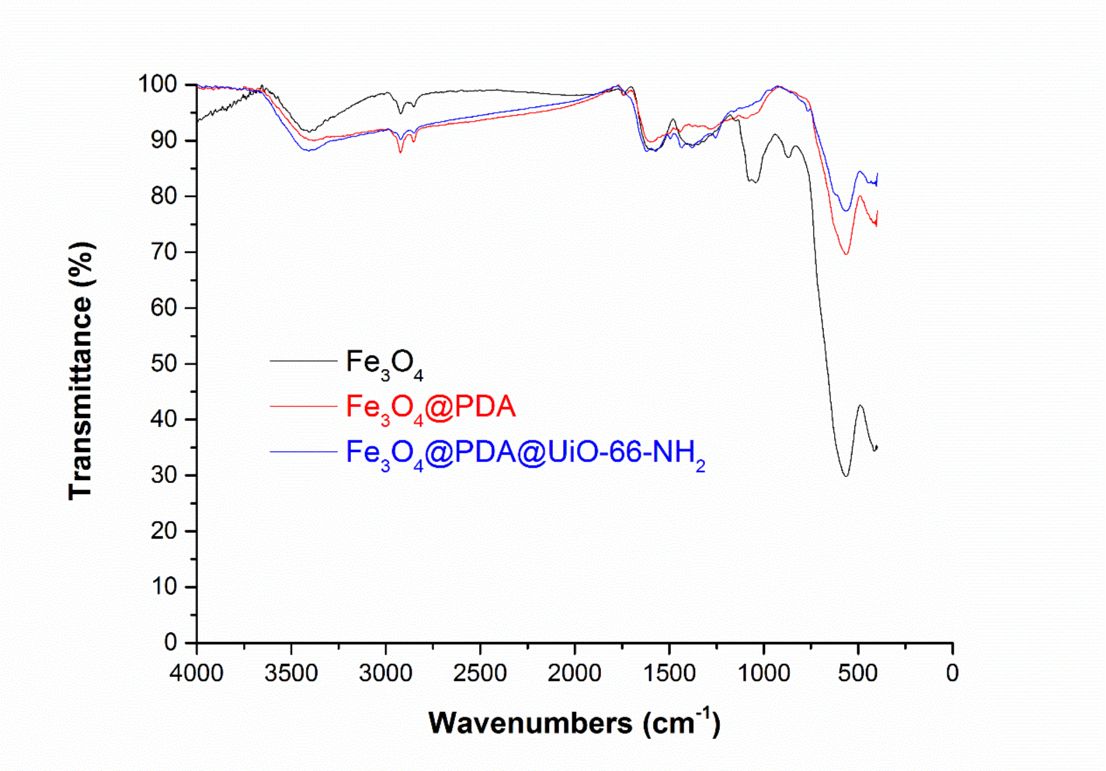

Figure S6. FT-IR spectra of Fe_3O_4, Fe_3O_4@PDA and Fe_3O_4@PDA@UiO-66-NH_2.

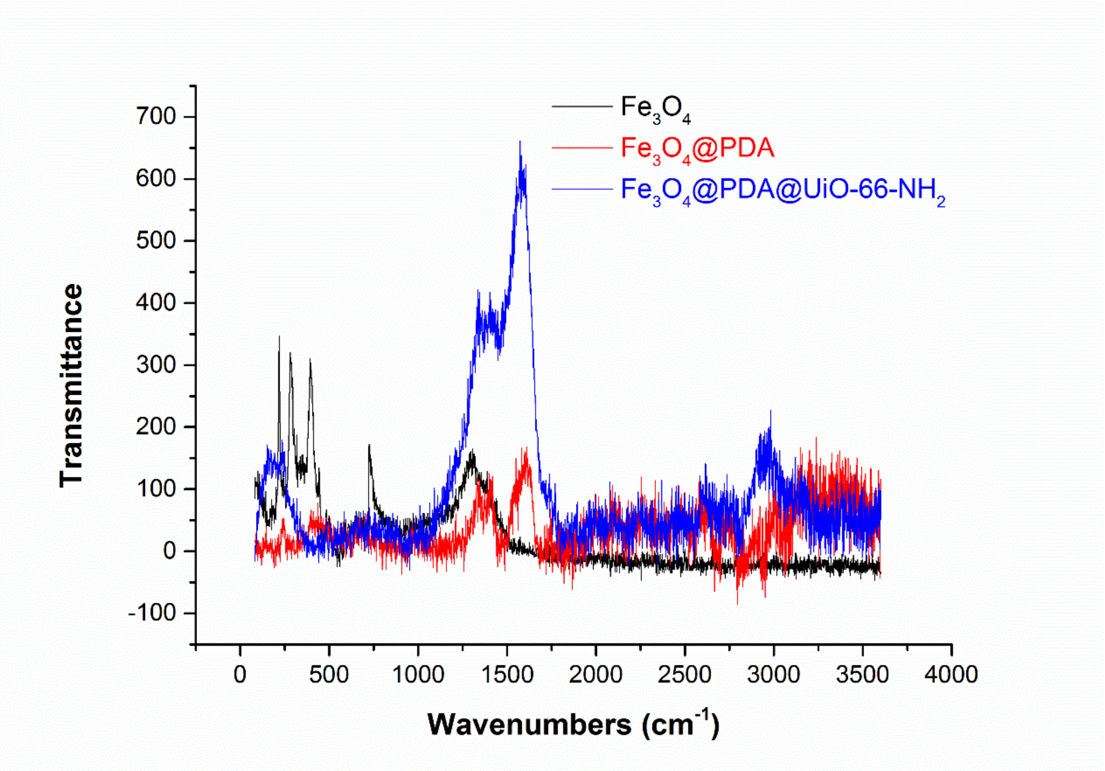

Figure S7. Raman spectra of Fe_3O_4, Fe_3O_4@PDA and Fe_3O_4@PDA@UiO-66-NH_2.

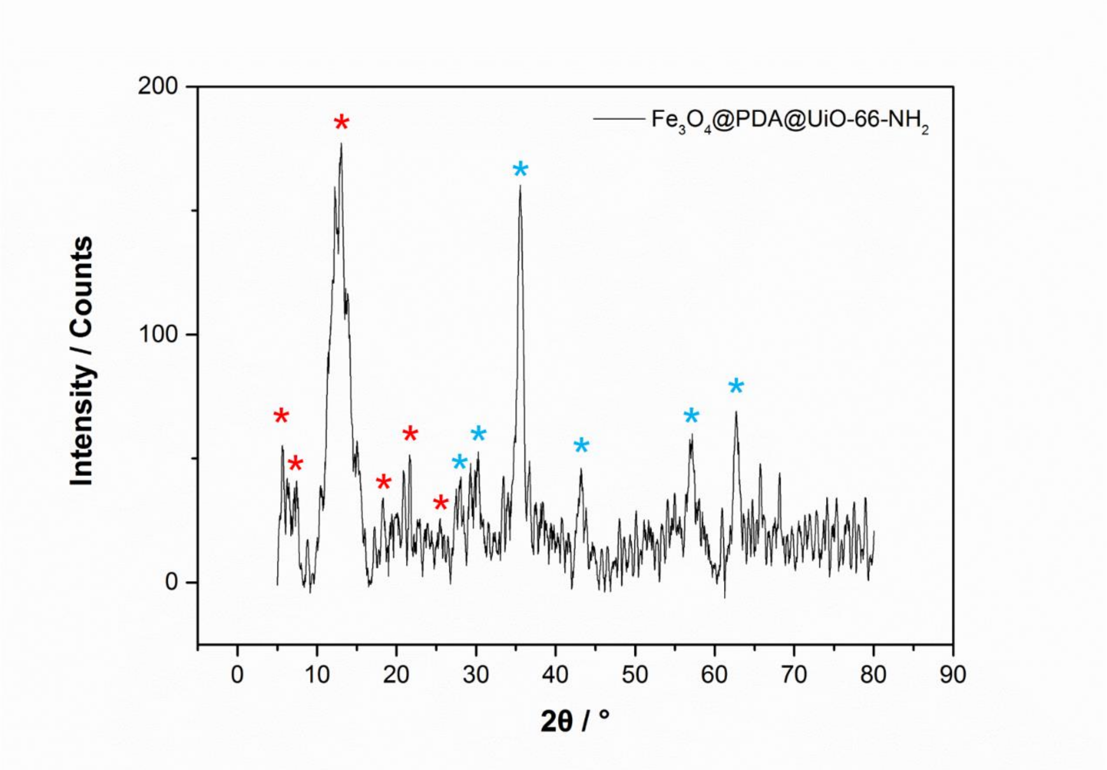

Figure S8. XRD patterns of Fe_3O_4@PDA@UiO-66-NH_2. Peaks from UiO-66-MOF were marked with * in red, while peaks from Fe_3O_4 were marked with * in blue.

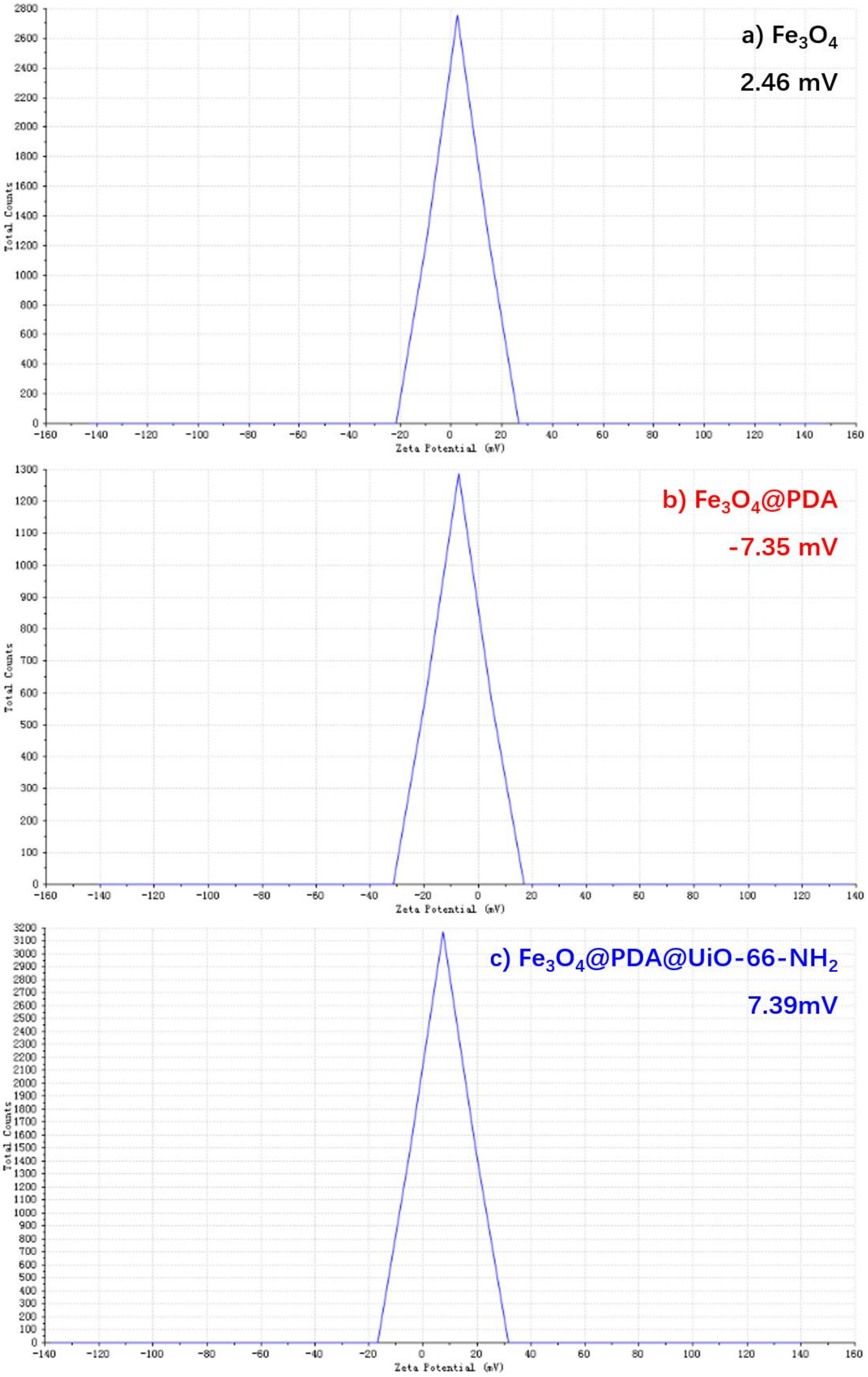

Figure S9. Zeta potential distributions of (a) Fe_3O_4, (b) Fe_3O_4@PDA and (c) Fe_3O_4@PDA@UiO-66-NH$_2$.

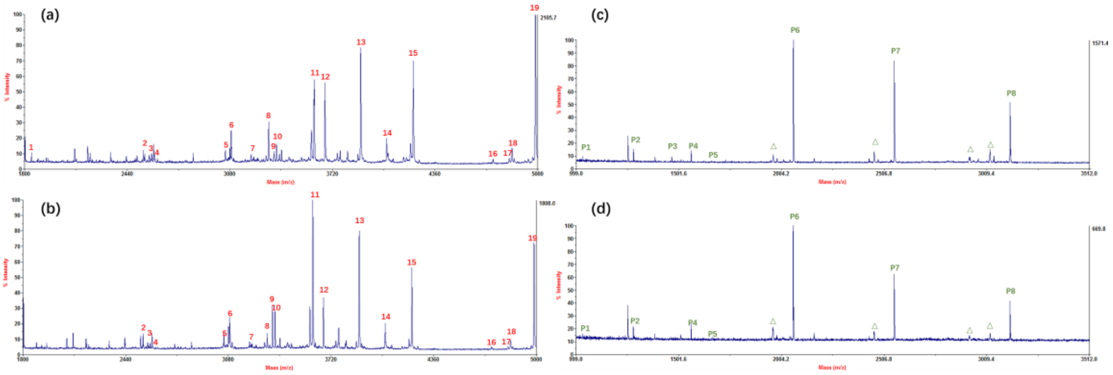

Figure S10. MALDI-TOF mass spectra for the glycopeptide enrichment from 250 fmol/μL HRP tryptic digest: (a) after treatment with the first-time Fe_3O_4@PDA@UiO-66-NH_2; (b) after treatment with the five-times-recycled Fe_3O_4@PDA@UiO-66-NH_2; for the phosphopeptide enrichment from 200 fmol/μL β-Casein tryptic digest: (c) after treatment with the first-time Fe_3O_4@PDA@UiO-66-NH_2; (d) after treatment with the five-times-recycled Fe_3O_4@PDA@UiO-66-NH_2, where glycopeptides were marked with Arabic Numerals in red, phosphopeptides were marked with Arabic Numerals in green, and △ indicates the losses of phosphoric acid.

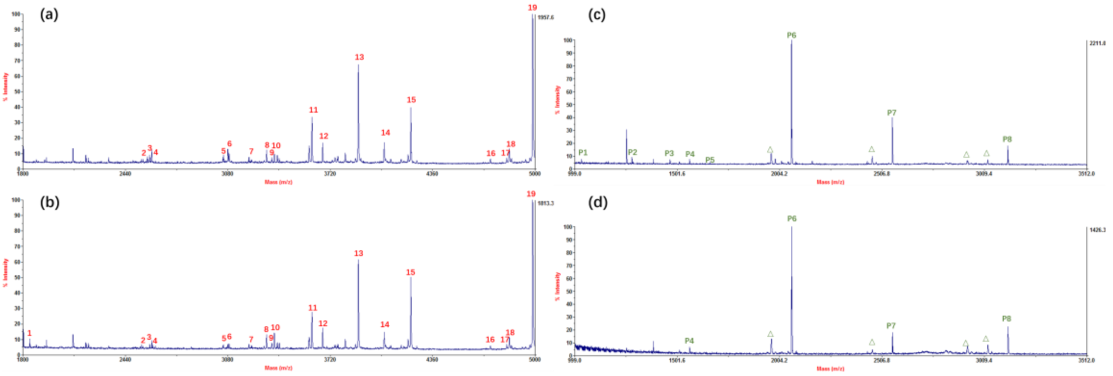

Figure S11. MALDI-TOF mass spectra for the glycopeptide enrichment from 250 fmol/μL HRP tryptic digest: (a) after treatment with the freshly-made Fe_3O_4@PDA@UiO-66-NH_2; (b) after treatment with the Fe_3O_4@PDA@UiO-66-NH_2 kept at -20C° for a month; for the phosphopeptide enrichment from 200 fmol/μL β-Casein tryptic digest: (c) after treatment with the freshly-made Fe_3O_4@PDA@UiO-66-NH_2; (d) after treatment with the Fe_3O_4@PDA@UiO-66-NH_2 kept at -20C° for a month, where glycopeptides were marked with Arabic Numerals in red, phosphopeptides were marked with Arabic Numerals in green, and △ indicates the losses of phosphoric acid.

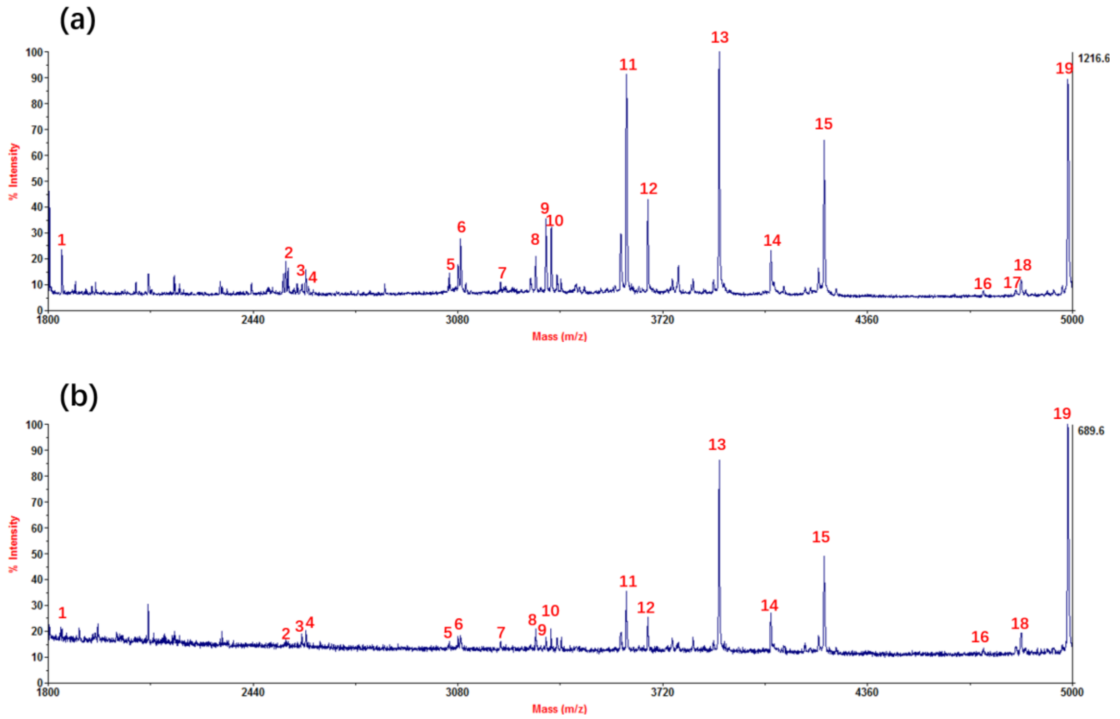

Figure S12. MALDI-TOF mass spectra for the glycopeptide enrichment from a mixture of HRP and BSA at a mass ratio of 1: 50: (a) after enrichment; 1: 100: (b) after enrichment, where glycopeptides were marked with Arabic Numerals in red.

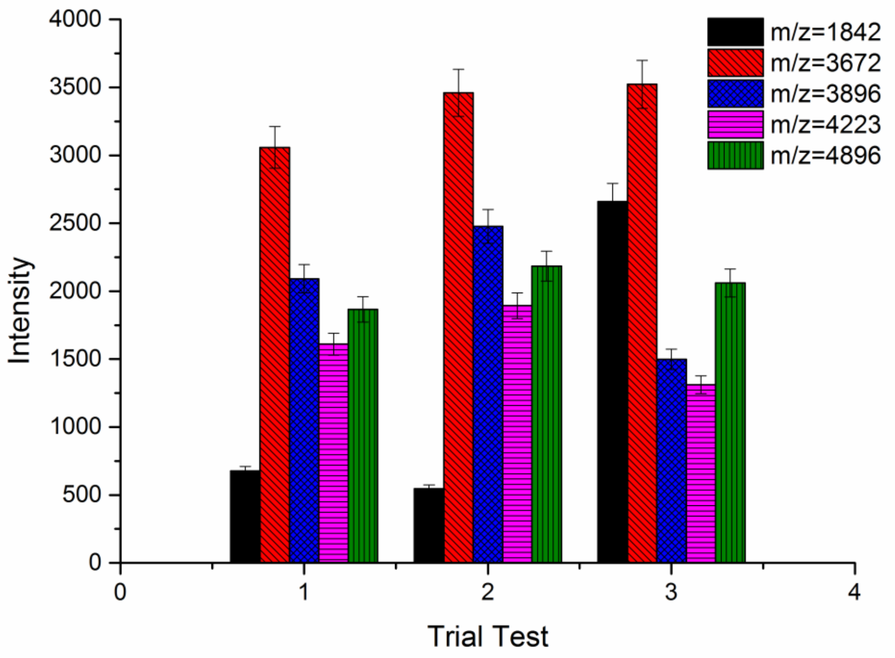

Figure S13. Recovery of the as-prepared MOFs through trial tests.

Table S1. Detailed information of glycopeptides identified from HRP digest.

Peak No.	Observed m/z	Glycan composition	Glycopeptide Sequence
1	1843.0	XylMan3FucGlcNAc2	NVGLN#R
2	2541.4	XylMan3FucGlcNAc2	SSPN#ATDTIPLVR
3	2591.4	XylMan3FucGlcNAc2	PTLN#TTYLQTLR
4	2611.4	XylMan3GlcNAc2	MGN#ITPLTGTQGQIR
5	3074.5	FucGlcNAc	LHFHDCFVNGCDASILLDN#TTSFR
6	3087.7	XylMan3FucGlcNAc2	GLCPLNGN#LSALVDFDLR
7	3222.9	Man3FucGlcNAc2	SFAN#STQTFFNAFVEAMDR
8	3321.8	XylMan3FucGlcNAc2	QLTPTFYDNSCPN#VSNIVR
9	3353.7	XylMan3FucGlcNAc2	SFAN#STQTFFNAFVEAMDR
10	3369.7	XylMan3FucGlcNAc2	SFAN#STQTFFNAFVEAM*DR
11	3605.0	XylMan3FucGlcNAc2	NQCRGLCPLNGN#LSALVDFDLR
12	3672.1	XylMan3FucGlcNAc2	GLIQSDQELFSSPN#ATDTIPLVR
13	3894.1	XylMan3FucGlcNAc2	LHFHDCFVNGCDASILLDN#TTSFR
14	4056.2	XylMan3GlcNAc2	QLTPTFYDNSC(AAVESACPR)PN#VSNIVR-H_2O
15	4222.4	XylMan3FucGlcNAc2	QLTPTFYDNSC(AAVESACPR)PN#VSNIVR
16	4719.6	Man3FucGlcNAc2, Man3FucGlcNAc2	LYN#FSNTGLPDPTLN#TTYLQTLR
17	4821.7	XylMan2FucGlcNAc2, XylMan2GlcNAc2	LYN#FSNTGLPDPTLN#TTYLQTLR
18	4838.7	XylMan3FucGlcNAc2, XylMan3GlcNAc2	LYN#FSNTGLPDPTLN#TTYLQTLR
19	4984.7	XylMan3FucGlcNAc2, XylMan3FucGlcNAc2	LYN#FSNTGLPDPTLN#TTYLQTLR

Table S2. Detailed information of glycopeptides identified from IgG digest.

Peak No.	Observed m/z	Glycan composition	Glycopeptide Sequence
I1	2399.3	[Hex]3[HexNAc]3[Fuc]1	EEQFN#STFR
I2	2431.3	[Hex]3[HexNAc]3[Fuc]1	EEQYN#STYR
I3	2457.3	[Hex]3[HexNAc]4	EEQFN#STFR
I4	2488.3	[Hex]3[HexNAc]4	EEQYN#STYR
I5	2561.4	[Hex]4[HexNAc]3[Fuc]1	EEQFN#STFR
I6	2602.4	[Hex]3[HexNAc]4[Fuc]1	EEQFN#STFR
I7	2618.4	[Hex]4[HexNAc]4	EEQFN#STFR
I8	2634.4	[Hex]3[HexNAc]4[Fuc]1	EEQYN#STYR
I9	2650.4	[Hex]3[HexNAc]4[Fuc]1	EEQYN#STYR
I10	2764.5	[Hex]4[HexNAc]4[Fuc]1	EEQFN#STFR
I11	2781.5	[Hex]5[HexNAc]4	EEQFN#STFR
I12	2796.5	[Hex]4[HexNAc]4[Fuc]1	EEQYN#STYR
I13	2805.5	[Hex]3[HexNAc]5[Fuc]1	EEQFN#STFR
I14	2837.5	[Hex]3[HexNAc]5[Fuc]1	EEQYN#STYR

I15	2853.5	[Hex]4[HexNAc]5	EEQYN#STYR
I16	2926.6	[Hex]5[HexNAc]4[Fuc]1	EEQFN#STFR
I17	2958.6	[Hex]5[HexNAc]4[Fuc]1	EEQYN#STYR
I18	2967.6	[Hex]4[HexNAc]5[Fuc]1	EEQFN#STFR
I19	3000.0	[Hex]4[HexNAc]5[Fuc]1	EEQYN#STYR
I20	3130.0	[Hex]5[HexNAc]5[Fuc]1	EEQFN#STFR
I21	3161.7	[Hex]5[HexNAc]5[Fuc]1	EEQYN#STYR

Table S3. Detailed information of phosphopeptides identified from β-Casein digest.

Peak No.	Observed m/z	Protein	Phosphopeptide Sequence
P1	1031.3785	β/33-48	FQpSEEQQQTEDELQDK
P2	1279.1156	β/33-52	FQpSEEQQQTEDELQDKIHPF
P3	1466.6918	α-S2/138-149	TVDMEpSTEVFTK
P4	1561.2365	β/1-25	RELEELNVPGEIVEpSLpSpSpSEESITR
P5	1660.9084	α-S1/106-119	VPQLEIVPNpSAEER
P6	2061.9438	β/33-48	FQpSEEQQQTEDELQDK
P7	2556.2473	β/33-52	FQpSEEQQQTEDELQDKIHPF
P8	3122.4907	β/1-25	RELEELNVPGEIVEpSLpSpSpSEESITR

Table S4. Detailed information of glycopeptides identified from human serum.

Peak No.	Protein Group Accessions	Sequence	MH+ [Da]
1		VTPAcnTSLPAQR	1415.69512
2		EIKNnQTEK	1104.55351
3		THTnISESHPnATFSAVGEASIcEDDWDSGER	3521.48655
4		DGQLLPSSnYSNIK	1536.75432
5		nISLQLmSNMNISnK	1724.81888
6	O00187	VVINSnITPIcLPR	1596.87810
7	O75882	ISnSSDTVEcEcSENWK	2045.80620
8		IDSTGnVTNELR	1319.64385
9		nHScSEGQISIFR	1535.69016
10		cInQSIcEK	1152.50102
11		GIcnSSDVR	1008.4411

			5
12		AATcINPLnGSVcERPAnHSAK	2369.09780
13	O95445	TELFSSScPGGIMLnETGQGYQR	2533.13578
14	O95497	LTGVAGnYTVcQK	1411.68999
15	P00450	EnLTAPGSDSAVFFEQGTTR	2127.98325
16		ELHHLQEQnVSNAFLDK	2022.99065
17		EHEGAIYPDnTTDFQR	1893.82634
18		ELHHLQEQnVSnAFLDK	2023.97275
19	P00734	SEGSSVnLSPPLEQcVPDR	2071.96416
20		GHVnITR	797.42632
21	P00736	cnYSIR	813.35637
22	P00738	VVLHPnYSQVDIGLIK	1795.99578
23		VVLHPnYSQVDIGLIKLK	2037.17557
24		QLVEIEKVVLHPnYSQVDIGLIK	2635.46718
25	P00739;P00738	NLFLnHSEnATAK	1460.70195
26		NLFLnHSENATAK	1459.71794
27		nLFLnHSEnATAK	1461.68694
28		MVSHHnLTTGATLINEQWLLTTAK	2680.37343
29		mVSHHnLTTGATLINEQWLLTTAK	2696.37734
30		mVSHHnLTTGATLInEQWLLTTAK	2697.37465
31	P00742	GDnnLTR	791.35204
32		GDNnLTR	790.36858
33	P00748	RnHScEPcQTLAVR	1728.79135
34	P00751	IVLDPSGSmNIYLVLDGSDSIGASnFTGAK	3058.48624
35	P01008	LGAcnDTLQQLMEVFK	1867.8914

			1
36		LGAcnDTLQQLmEVFK	1883.89153
37		SLTFnETYQDISELVYGAK	2179.04551
38		LGAcnDTLQQLMEVFKFDTISEK	2688.28983
39		WVSnKTEGR	1077.53226
40	P01009	YLGnATAIFFLPDEGK	1756.88042
41		ADTHDEILEGLNFnLTEIPEAQIHEGFQELLR	3692.80849
42	P01011	TLnQSSDELQLSMGNAMFVK	2214.04546
43		YTGnASALFILPDQDK	1753.86528
44		FnLTETSEAEIHQSFQHLLR	2401.17972
45		TLnQSSDELQLSmGNAMFVK	2230.03617
46		LInDYVKnGTR	1294.66448
47	P01019	LQAILGVPWKDKncTSR	1987.04129
48		DKncTSR	881.37828
49	P01023	GcVLLSYLnETVTVSASLESVR	2398.22099
50		VSnQTLSLFFTVLQDVPVR	2164.16435
51		SLGNVnFTVSAEALESQELcGTEVPSVPEHGR	3414.61521
52		SLGNVnFTVSAEALESQELcGTEVPSVPEHGRK	3542.71157
53		SLGnVnFTVSAEALESQELcGTEVPSVPEHGR	3415.61367
54		SLGnVnFTVSAEALESQELcGTEVPSVPEHGRK	3543.71555
55	P01024	TVLTPATNHMGnVTFTIPANR	2256.14499
56		TVLTPATNHmGnVTFTIPANR	2272.14263
57		TVLTPATnHMGnVTFTIPANR	2257.12827

58		TVLTPATnHmGnVTFTIPANR	2273.11832
59	P01033	FVGTPEVnQTTLYQR	1753.87956
60	P01042	YNSQnQSNNQFVLYR	1875.86357
61		LnAENnATFYFK	1433.65801
62		LNAENnATFYFK	1432.67473
63	P01591	EnISDPTSPLR	1229.60124
64		IIVPLNNREnISDPTSPLR	2149.15885
65		IIVPLnNREnISDPTSPLR	2150.14560
66	P01833	AnLTNFPENGTFVVnIAQLSQDDSGR	2809.32822
67		LSLLEEPGnGTFTVILNQLTSR	2403.27445
68		AnLTnFPEnGTFVVNIAQLSQDDSGR	2810.33159
69	P01857	EEQYnSTYR	1190.49529
70		TKPREEQYnSTYR	1672.79294
71	P01859	EEQFnSTFR	1158.50640
72		TKPREEQFnSTFR	1640.80295
73	P01860	EEQYnSTFR	1174.50115
74	P01861	EEQFnSTYR	1174.50139
75	P01871	GLTFQQnASSMcVPDQDTAIR	2340.05303
76		STGKPTLYnVSLVMSDTAGTcY	2366.08769
77		GLTFQQnASSmcVPDQDTAIR	2356.05864
78		THTnISESHPnATFSAVGEASIcEDDWNSGER	3520.48330
79		nNSDISSTR	994.44359
80		STGKPTLYnVSLVmSDTAGTcY	2382.0855

			0
81		THTnISESHPnATFSAVGEASIcEDDWnSGER	3521.48655
82	P01876	LAGKPTHVnVSVVmAEVDGTcY	2364.12212
83		LAGKPTHVnVSVVMAEVDGTcY	2348.12656
84	P01876;P01877	LSLHRPALEDLLLGSEAnLTcTLTGLR	2964.58217
85	P01877	TPLTAnITK	959.54088
86	P02743	ESVTDHVNLITPLEKPLQnFTLcFR	2972.52065
87	P02748	AVnITSENLIDDVVSLIR	1972.06059
88	P02749	VYKPSAGnNSLYR	1469.73804
89		LGnWSAmPScK	1267.54424
90		LGnWSAMPScK	1251.55034
91		DTAVFEcLPQHAMFGnDTITcTTHGnWTK	3354.46157
92		DTAVFEcLPQHAmFGnDTITcTTHGnWTK	3370.44765
93	P02750	mFSQnDTR	1015.41484
94		LPPGLLAnFTLLR	1425.84856
95	P02751	LDAPTNLQFVnETDSTVLVR	2233.14090
96		DQcIVDDITYNVnDTFHK	2197.97246
97		NSITLTnLTPGTEYVVSIVALnGR	2534.35698
98	P02763	QDQcIYnTTYLNVQR	1916.88140
99		QDQcIYnTTYLnVQR	1917.86833
100		EnGTISR	777.37401
101	P02765	KVcQDcPLLAPLnDTR	1900.92070
102		AALAAFNAQNnGSNFQLEEISR	2366.13872
103		AALAAFNAQnnGSNFQLEEISR	2367.1272

			4
104		VcQDcPLLAPLnDTR	1772.83391
105	P02774	LcDnLSTK	951.44542
106	P02786	DFEDLYTPVnGSIVIVR	1937.98772
107		KDFEDLYTPVnGSIVIVR	2066.08243
108	P02787	QQQHLFGSnVTDcSGNFcLFR	2516.10990
109		QQQHLFGSnVTDcSGnFcLFR	2517.09464
110	P02790	ALPQPQnVTSLLGcTH	1736.86382
111		SWPAVGncSSALR	1405.65325
112		cSDGWSFDATTLDDnGTMLFFK	2529.05742
113		LNAAKALPQPQnVTSLLGcTH	2234.16355
114		nGTGHGnSTHHGPEYmR	1869.75820
115	P03951	LETTVnYTDSQRPIcLPSK	2223.09770
116		VYSGILnQSEIK	1351.71147
117		LETTVnYTDSQRPIcLPSKGDR	2551.24509
118		GINYnSSVAK	1053.52092
119	P03952	IYPGVDFGGEELnVTFVK	1984.99004
120		IYSGILnLSDITK	1437.78386
121		LQAPLnYTEFQKPIcLPSK	2248.16850
122		IVGGTnSSWGEWPWQVSLQVK	2359.17266
123		GVNFnVSK	865.44145
124	P04003	LSVDKDQYVEPEnVTIQcDSGYGVVGPQSITcSGnR	3973.81528
125		LSVDKDQYVEPEnVTIQcDSGYGVVGPQSITcSGNR	3972.82688
126	P04004	NnATVHEQVGGPSLTSDLQAQSK	2382.1563

			5
127		nnATVHEQVGGPSLTSDLQAQSK	2383.14420
128	P04070	EVFVHPnYSK	1220.59477
129	P04114	FnSSYLQGTNQITGR	1686.80852
130		FnSSYLQGTnQITGR	1687.79643
131		FVEGSHnSTVSLTTK	1607.79167
132		YDFnSSMLYSTAK	1527.66765
133		QVLFLDTVYGncSTHFTVK	2230.08030
134		YDFnSSmLYSTAK	1543.66118
135		FEVDSPVYnATWSASLK	1914.91838
136		QVFPGLNYcTSGAYSnASSTDSASYYPLTGDTR	3551.55722
137		IQSPLFTLDAnADIGnGTTSANEAGIAASITAK	3234.62656
138	P04180	AELSnHTRPVILVPGcLGNQLEAK	2617.37636
139	P04196	VIDFncTTSSVSSALANTK	2015.95976
140		VIDFncTTSSVSSALAnTK	2016.94365
141	P05090	ADGTVNQIEGEATPVnLTEPAK	2255.11235
142		ADGTVnQIEGEATPVnLTEPAK	2256.08940
143		ADGTVNQIEGEATPVnLTEPAKLEVK	2724.39438
144		cIQAnYSLMENGK	1528.67742
145		ADGTVnQIEGEATPVnLTEPAKLEVK	2725.37601
146	P05155	VLSnNSDANLELINTWVAK	2102.07891
147		DTFVnASR	910.42650
148		VGQLQLSHnLSLVILVPQNLK	2314.34928

149	P05156	FLNnGTcTAEGK	1312.58391
150		FLnnGTcTAEGK	1313.56841
151		LSDLSInSTEcLHVHcR	2041.94497
152		nGTAVcATNR	1064.47954
153		nGTAVcATNRR	1220.58030
154	P05160	EHETcLAPELYnGnYSTTQK	2357.02622
155		EHETcLAPELYNGnYSTTQK	2356.03800
156	P05362	AnLTVVLLR	999.61992
157		LNPTVTYGnDSFSAK	1614.76579
158	P05543	VTAcHSSQPnATLYK	1677.79045
159		TLYETEVFSTDFSnISAAK	2124.00395
160	P05546	DFVnASSKYEITTIHNLFR	2256.12661
161		nLSMPLLPADFHK	1483.76108
162		DFVnASSK	868.40434
163	P06276	EnETEIIK	976.48265
164		DnYTKAEEILSR	1439.70069
165		DnNSIITR	933.46343
166	P06681	QSVPAHFVALnGSK	1455.75957
167		LTDTIcGVGnMSAnASDQER	2140.90923
168		LGSYPVGGnVSFEcEDGFILR	2317.08965
169		LTDTIcGVGnmSAnASDQER	2156.90776
170		TmFPnLTDVR	1210.57744
171	P07602	TnSTFVQALVEHVKEEcDR	2263.06630
172	P07996	VVnSTTGPGEHLR	1367.69153

173		VScPIMPcSnATVPDGEccPR	2407.98447
174	P08185	AVLQLNEEGVDTAGSTGVTLnLTSKPIILR	3110.69272
175		AQLLQGLGFnLTER	1560.83769
176	P08603	ISEEnETTcYMGK	1562.63567
177		MDGASnVTcINSR	1425.61040
178		IPcSQPPQIEHGTInSSR	2021.97136
179		ISEEnETTcYmGK	1578.62908
180		MDGASnVTcInSR	1426.59307
181		SPDVInGSPISQK	1342.68547
182	P0C0L5;P0C0L4	FSDGLESnSSTQFEVK	1775.79802
183		nTTcQDLQIEVTVK	1649.80974
184		GLnVTLSSTGRnGFK	1552.79692
185		FSDGLESnSSTQFEVKK	1903.89690
186		GLnVTLSSTGR	1105.58501
187	P10643	nYTLTGR	825.40990
188	P10721	SEnESNIR	949.42266
189	P10909	LAnLTQGEDQYYLR	1684.81828
190		mLnTSSLLEQLNEQFNWVSR	2426.17095
191		MLnTSSLLEQLNEQFNWVSR	2410.17057
192		KKEDALnETR	1204.61760
193		KEDALnETR	1076.52214
194		EDALnETR	948.42717
195		EDALnETRESETK	1522.68718
196		ELPGVcnETMMALWEEcKPcLK	2696.1917

			5
197		HnSTGcLR	945.42052
198	P11362	HIEVnGSK	884.44713
199	P11597	SIDVSIQnVSVVFK	1535.83122
200	P12259	TNInSSR	792.38402
201		NSVLnSSTAEHSSPYSEDPIEDPLQPDVTGIR	3455.60959
202	P13473	VASVININPnTTHSTGScR	2028.97807
203		LnSSTIK	763.41954
204	P13598	GnETLHYETFGK	1396.63921
205	P13671	VLnFTTK	823.45543
206		LSSnSTK	737.36778
207	P14151	DnYTDLVAIQNK	1394.67888
208		FcRDnYTDLVAIQNK	1857.87998
209	P15144	nATLVNEADKLR	1344.71172
210	P17936	AYLLPAPPAPGnASESEEDR	2084.97856
211		GLcVnASAVSR	1134.55742
212		VDYESQSTDTQnFSSESKR	2208.94754
213	P19652	QNQcFYnSSYLNVQR	1921.85100
214		QnQcFYnSSYLNVQR	1922.83574
215		EnGTVSR	763.35808
216		EnGTVSRYEGGR	1325.60840
217	P19823	GAFISnFSMTVDGK	1474.68865
218		GAFISnFSmTVDGK	1490.68376
219	P20851	LGHcPDPVLVNGEFSSSGPVnVSDK	2612.23258
220		LGHcPDPVLVnGEFSSSGPVnVSDK	2613.21109
221		EWDnTTTEcR	1312.51116

222		TLFcnASK	941.43877
223		TLFcnASKEWDnTTTEcR	2234.93058
224		KTLFcnASK	1069.53348
225	P22792	LYLGSNnLTALHPALFQnLSK	2316.22002
226		AFGSNPnLTK	1049.52641
227		LYLGSnnLTALHPALFQnLSK	2317.20957
228	P23142	cATPHGDnASLEATFVK	1818.83127
229	P25311	DIVEYYNDSnGSHVLQGR	2066.94853
230		DIVEYYnDSnGSHVLQGR	2067.92803
231	P26927	GTAnTTTAGVPcQR	1434.66619
232		GTGnDTVLNVALLNVISNQEcNIK	2587.30368
233	P27169	VTQVYAEnGTVLQGSTVASVYK	2315.17851
234		HAnWTLTPLK	1181.63042
235	P29622	SQILEGLGFnLTELSESDVHR	2345.16094
236		FLnDTMAVYEAK	1402.65642
237		DFYVDEnTTVR	1359.60808
238	P35858	AGAFLGLTNVAVmnLSGNcLR	2195.10210
239		AGAFLGLTNVAVMnLSGNcLR	2179.10025
240	P36955	VTQnLTLIEESLTSEFIHDIDR	2574.29033
241	P36980	LQNNENnIScVER	1590.71794
242		LQNNEnnIScVER	1591.70366
243	P40197	LLDLSGNnLTHLPK	1535.84250
244	P41222	SVVAPATDGGLnLTSTFLR	1920.0063

			8
245	P43251	YQFNTNVVFSNnGTLVDR	2088.99883
246		NPVGLIGAEnATGETDPSHSK	2094.99424
247		FnDTEVLQR	1122.54326
248		DVQIIVFPEDGIHGFnFTR	2205.09830
249	P43652	YAEDKFnETTEK	1475.65764
250		DIENFnSTQK	1196.54338
251		nccNTENPPGcYR	1642.60710
252		DIEnFnSTQK	1197.52593
253		FnETTEK	869.38860
254	P48740	NnLTTYK	854.42546
255	P49908	EGYSnISYIVVNHQGISSR	2124.03667
256	P51884	LHINHNnLTESVGPLPK	1883.99741
257		AFEnVTDLQWLILDHNLLENSK	2613.31931
258		LSHNELADSGIPGnSFnVSSLVELDLSYNK	3221.57370
259	P55058	VSnVScQASVSR	1294.60539
260		EGHFYYnISEVK	1486.68559
261		IYSnHSALESLALIPLQAPLK	2279.26444
262	P55103	EQEcEIISFAETGLSTInQTR	2427.13565
263	P55290	InNTHALVSLLQNLNK	1792.99179
264	P80108	LGTSLSSGHVLMnGTLK	1715.90080
265		nLTTSLTESVDR	1336.65788
266		NInYTER	910.42656
267		LGTSLSSGHVLmnGTLK	1731.89463

268	Q06033	NAHGEEKEnLTAR	1469.69822
269		EnLTAR	704.35698
270	Q08380	TVIRPFYLTnSSGVD	1669.84392
271		ALGFEnATQALGR	1348.68511
272		GLnLTEDTYKPR	1407.71184
273		DAGVVcTnETR	1222.53728
274	Q12913	TPEQGSnGTDGASQK	1477.64104
275		IHVAGETDSSNLnVSEPR	1925.92124
276	Q12981	AnLTcK	707.33843
277	Q13201	LnDSIQTLVNDNQR	1630.80327
278		FNPGAESVVLSnSTLK	1663.85613
279		VnESVVSIAAQQK	1373.72759
280		LQnLTLPTnASIK	1414.77715
281		KIDnISLTVNDVR	1487.80461
282	Q14624	LPTQnITFQTESSVAEQEAEFQSPK	2810.33476
283	Q15063	EVnDTLLVNELK	1387.73406
284	Q6EMK4	LHEITnETFR	1260.62224
285	Q6UXB8	SLPNFPnTSATAnATGGR	1777.83623
286	Q6YHK3	TQDEILFSnSTR	1411.66924
287	Q7Z7G0	EnGSFSGK	826.35741
288	Q8IZF2	YEEQQLEIQnSSR	1624.74443
289	Q92954	nGTLVAFR	878.47283
290	Q96IY4	QVHFFVnASDVDNVK	1719.83464
291	Q96KN2	LVPHmnVSAVEK	1340.68791

292		LVPHMnVSAVEK	1324.69361
293	Q96PD5	LEPVHLQLQcmSQEQLAQVAAnATK	2824.39908
294		LEPVHLQLQcMSQEQLAQVAAnATK	2808.40317
295		GFGVAIVGnYTAALPTEAALR	2092.10758
296	Q9BY67	FQLLnFSSSELK	1413.72649
297	Q9HDC9	AGPnGTLFVADAYK	1424.70464
298	Q9NQ38	nGTLIcTR	935.46178
299	Q9UGM5	YNNEnTSK	970.40746
300	Q9UK55	LPYQGnATMLVVLMEK	1807.93218
301		ETFFnLSK	986.48314
302		ETFFnLSKR	1142.58415
303	Q9Y5Y7	KANQQLnFTEAK	1392.71147
304		ANQQLnFTEAK	1264.61699
305	Q9Y6R7	YLPVnSSLLTSDcSER	1841.85710
306		VVTVAALGTnISIHKDEIGK	2066.14931
307		LLISSLSESPASVSILSQADnTSK	2448.27829

Table S5. Detailed information of phosphopeptides identified from human serum.

Peak No.	Observed m/z	Phosphopeptide
**1**	1389.7030	ADpSGEGDFLAEGGGV
**2**	1460.7516	DpSGEGDFLAEGGGV
**3**	1545.8314	DpSGEGDFLAEGGGVR
**4**	1616.8799	ADpSGEGDFLAEGGGVR

Table S6. Detailed information of phosphopeptides identified from human serum.

Peak No.	Protein Group Accessions	Sequence	MH+ [Da]
1		AAFEcMyTLLDscLDR	2124.78476
2		GEQGDGLRELNKQEsASDmTSTFPVAQsLtPGsMEER	4318.72702
3		NLFHFGEsTtGsNFSFK	2159.79253

4		NVyMLAttVSSK	1553.57512
5		QQQQDsIDPsSR	1548.57584
6		RtVLTTQPNGLTTVGK	1765.92363
7		tFsFAIPLIEK	1425.64286
8		tVVsSPGPGSGPGPGTTsGAssPARPAtPLVPcR	3639.39145
9	A6NKC4	tNISHNGTYHcSGK	1655.65337
10	O14791	VTEPIsAESGEQVER	1710.76006
11	O95294	AKssSLNVR	1121.47209
12	P00747	QLGAGsIEEcAAK	1413.60808
13	P01008	ATEDEGsEQKIPEATNR	1954.83891
14	P01042	DIPTNsPELEETLTHTITK	2219.04856
15		DIPTNsPELEETLTHTITK	2219.03647
16		EsNEELTEScETK	1635.61089
17		ETTcSKEsNEELTEScETK	2341.90093
18		ETtcSKESNEELTEScETK	2341.90702
19		ETTcSKEsNEELTEScETKK	2469.99826
20	P02649	GEVQAMLGQsTEELR	1727.77031
21	P02751	TNTNVNcPIEcFMPLDVQADREDsRE	3190.33152
22	P02765	cDSSPDsAEDVR	1417.49126
23		cDSSPDsAEDVRK	1545.59490
24	P02768	ADDKEtcFAEEGKK	1707.68852
25		EtYGEMADccAK	1514.49040
26		TcVADEsAENcDK	1578.54607
27	P04114	VREsDEETQIK	1413.62639
28	P05546	ENtVTNDWIPEGEEDDDYLDLEK	2819.13132
29		GGEtAQSADPQWEQLNNK	2052.86724
30	P10909	VtTVASHTSDSDVPSGVTEVVVK	2394.14444
31	P49908	DMPAsEDLQDLQK	1569.65190
32	Q05084	TEDGKSILsALDK	1456.69768
33	Q9H5V7	tTPTGGLPR	979.46288

References

1 Sun, S. & Zeng, H. Size-Controlled Synthesis of Magnetite Nanoparticles. *J. Am. Chem. Soc.* **124**, 8204-8205 (2002).
